# CRC-113 gene expression signature for predicting prognosis in patients with colorectal cancer

**DOI:** 10.18632/oncotarget.5183

**Published:** 2015-09-11

**Authors:** Minh Nam Nguyen, Tae Gyu Choi, Dinh Truong Nguyen, Jin-Hwan Kim, Yong Hwa Jo, Muhammad Shahid, Salima Akter, Saurav Nath Aryal, Ji Youn Yoo, Yong-Joo Ahn, Kyoung Min Cho, Ju-Seog Lee, Wonchae Choe, Insug Kang, Joohun Ha, Sung Soo Kim

**Affiliations:** ^1^ Department of Biochemistry and Molecular Biology, Medical Research Center for Bioreaction to Reactive Oxygen Species and Biomedical Science Institute, School of Medicine, Kyung Hee University, Seoul, Republic of Korea; ^2^ School of Biotechnology, Tan Tao University, Long An, Vietnam; ^3^ Department of Systems Biology, Division of Cancer Medicine, The University of Texas MD Anderson Cancer Center, Houston, Texas, USA

**Keywords:** colorectal cancer, microarray analysis, gene expression profile, risk prediction

## Abstract

Colorectal cancer (CRC) is the third leading cause of global cancer mortality. Recent studies have proposed several gene signatures to predict CRC prognosis, but none of those have proven reliable for predicting prognosis in clinical practice yet due to poor reproducibility and molecular heterogeneity. Here, we have established a prognostic signature of 113 probe sets (CRC-113) that include potential biomarkers and reflect the biological and clinical characteristics. Robustness and accuracy were significantly validated in external data sets from 19 centers in five countries. In multivariate analysis, CRC-113 gene signature showed a stronger prognostic value for survival and disease recurrence in CRC patients than current clinicopathological risk factors and molecular alterations. We also demonstrated that the CRC-113 gene signature reflected both genetic and epigenetic molecular heterogeneity in CRC patients. Furthermore, incorporation of the CRC-113 gene signature into a clinical context and molecular markers further refined the selection of the CRC patients who might benefit from postoperative chemotherapy. Conclusively, CRC-113 gene signature provides new possibilities for improving prognostic models and personalized therapeutic strategies.

## INTRODUCTION

Colorectal cancer (CRC) is one of the leading causes of morbidity and mortality in the world. It is the third most common cause of death worldwide, accounting for 8% of all cancer-related deaths [1–3]. The American Joint Committee on Cancer (AJCC) staging system is the current standard for determining patient prognosis [4]. Usually, stage II and stage III patients at risk of locoregional or distant relapse are designated for chemotherapy while stage I patients are cured by surgery only [5]. However, pathological staging fails to accurately predict recurrence in many patients undergoing curative surgery for localized CRC, because CRC is a highly heterogeneous disease [6]. Practically, 10–20% of patients with stage II CRC, and 30–40% of those with stage III CRC develop recurrence [7]. Thus, molecular markers have extensively been investigated for CRC characterization and prognosis. Microsatellite instability (MSI), caused by defective function of the DNA mismatch repair (MMR) system, has reproducibly been found to constitute a significant prognostic factor in early CRC in both a meta-analysis and prospective trials [8–10]. KRAS is also a reliable predictive marker in EGFR-targeted therapies of advanced CRC [11, 12]. Other DNA alterations, chromosomal instability (CIN), CpG island methylator phenotype (CIMP), p53 and BRAF should be further defined for reproducible molecular classification [11, 12].

Gene expression profiling has shown great promise in predicting prognosis of individual patients in diverse cancers. Several gene signatures have thus been developed to classify various prognostic groups beyond the CRC clinicopathological features. However, no signature has been clinically reliable yet. This poor reproducibility is attributed to the heterogeneity that develops in CRC through the integration of genetic and epigenetic features [13–16]. Therefore, it is critically required to establish a prognostic gene signature that would reflect the molecular heterogeneity of CRC in both genetic and epigenetic aspects, and would be used clinically to accurately predict recurrence risk and guide decisions of adjuvant therapy for the patients.

In this study, we established a novel prognostic gene signature to distinguish low and high risk patients using a gene expression profiling technique in six independent data sets from 19 centers in five countries. Then, we assessed the associations between the gene signature, clinicopathological factors and molecular alterations. We further investigated whether the new gene signature would help to develop adjuvant therapeutic strategies for stage III CRC patients. Finally, we attempted to provide possibilities for improving prognostic models of CRC heterogeneous aspects.

## RESULTS

### CRC-113 gene signature

**Figure 1:**
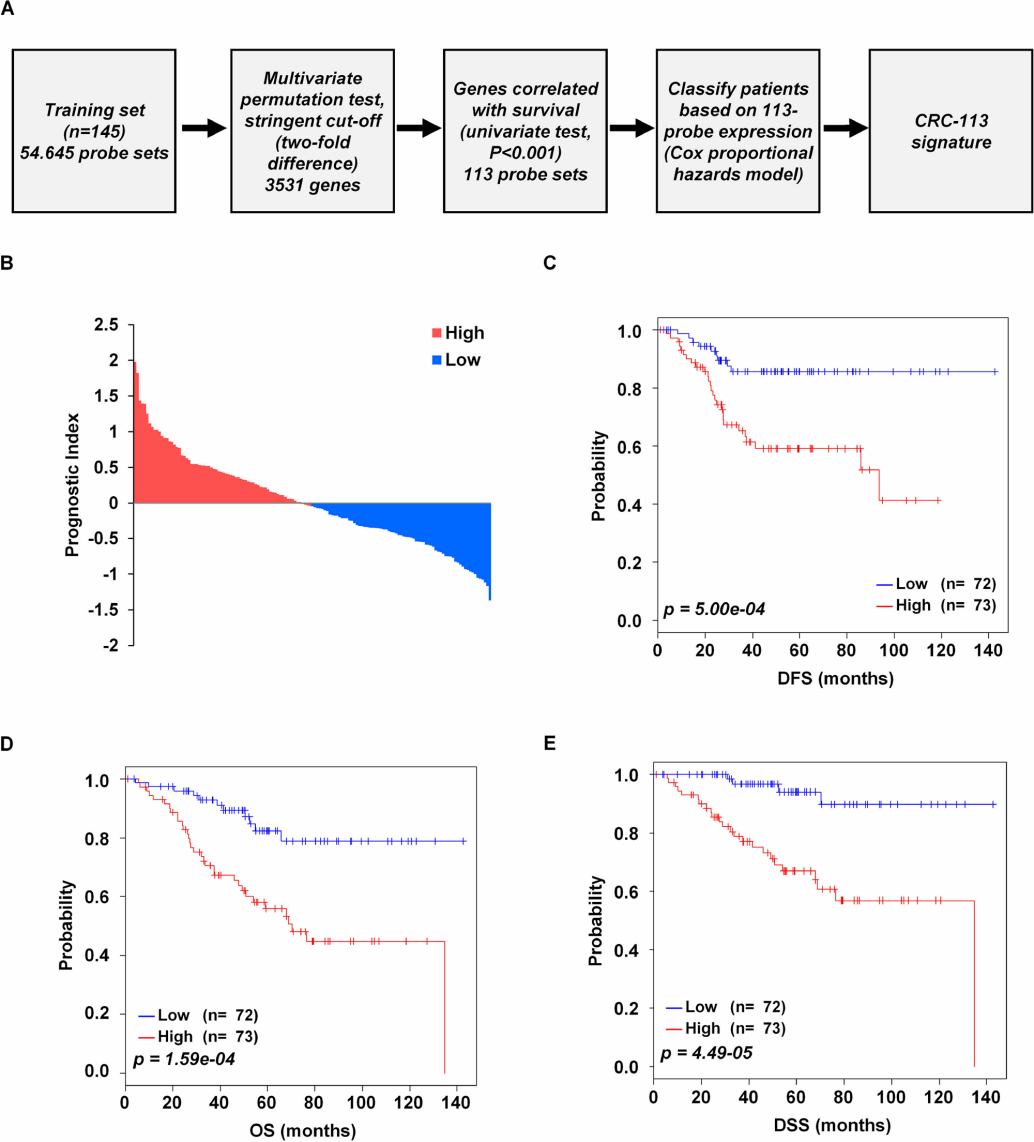
Survival analysis of the discovery data set **A.** Schematic overview of the procedure used to construct CRC-113 gene signature based on gene expression data. **B.** The relative prognostic index based on CRC-113 gene signature expression of each patient. The weight of each gene was calculated by the Cox proportional hazard regression model. **C–E.** Kaplan-Meier plots for DFS, OS, and DSS of two risk groups in the discovery data set. *p* values were computed by log-rank test.

In order to generate a molecular classifier that distinguishes low and high risk patients, gene expression profilings were analyzed in relation to survival data. We used the GSE17538 data set as a discovery data set [17, 18]. After filtering for probe set intensity, 3531 probe sets were analyzed in a univariate Cox regression analysis with DFS as the survival end point, *as discussed previously* [19, 20]. As a result, the gene signature with 113 probe sets was developed, and shown to be associated with DFS (false discovery rate of <10%). This model was termed the CRC-113 gene signature. A flow chart of the procedure used to generate the gene signature was provided (Figure 1A). Prognostic index for each patient was calculated based on the CRC-113 gene signature (Figure 1B). The patients were classified into high (*n* = 73) and low (*n* = 72) risk groups by risk relied on their prognostic index. Survival differences between predicted low and high risk outcome groups were evaluated with Kaplan-Meier survival curves for each follow-up time: DFS (*p* = 5.00e-04; Figure 1C), OS (*p* = 1.59e-04; Figure 1D), and DSS (*p* = 4.49e-05; Figure 1E) of patients classified by CRC-113 gene signature. A positive weighting coefficient indicates that the increased expression contributes to the high value for the CRC-113 gene signature value and thus a higher risk for poor survival. The 113 probe sets corresponded to 77 annotated genes (24 genes represented by more than one probe set), one expressed sequence tag clone, and two probe sets have no annotation (Supplementary Table S1). The resultant expression patterns of CRC-113 gene signature presented the low and high risk patient groups into two clusters (Supplementary Figure S1). Several independent studies previously proposed different gene signatures to identify CRC subtypes for predicting prognosis. We thus investigated whether the genes in CRC-113 gene signature were overlapped with those in the reported signatures: 21 genes (30 probes), 36 genes (53 probes) and 17 genes (17 probes) were in common with those published in the validation data sets from Jorrisen, *et al.* (GSE14333) [21], De Sousa E Melo, et al. (GSE33113) [22], Marisa, et al. (GSE39582) [23], respectively, and 13 genes (17 probes) in Oh, *et al.* [24]. However, there was no common probe in the probe sets from another study of De Sousa E Melo, et al. [25].

### CRC-113 gene signature and clinical relevance

To investigate the association between the CRC-113 gene signature classifier and clinicopathological characteristics, including gender, age at diagnosis, AJCC disease stage, grade, race and each follow-up time, we performed Chi-square (χ^2^) test (Table 1). The AJCC stage (*p* = 8.45e-03) and patient follow-up times (DFS, *p* = 6.43e-04; OS, *p* = 1.79e-04; DSS, *p* = 3.74e-04, respectively) were significantly correlated to our classification, while the others were not associated. To compare the prognostic value of our CRC-113 gene signature with other prognostic covariates, we performed univariate and multivariate Cox regression analysis using the discovery data set (Table 2). In univariate analysis, AJCC stage was significantly associated with DFS (HR 2.0, 95% CI 1.4–3.1, *p* = 7.24e-04), OS (HR 1.8, 95% CI 1.3–2.6, *p* = 1.82e-03) and DSS (HR 2.1, 95% Cl 1.3–3.8, *p* = 2.77e-03). The AJCC stage remained significantly associated with patient prognosis in DFS (HR 1.9, 95% CI 1.2–2.9, *p* = 5.29e-03), OS (HR 1.9, 95% CI 1.2–2.9, *p* = 5.38e-03) and DSS (HR 2.3, 95% Cl 1.2–4.2, *p* = 0.011) in multivariate analysis. Notably, CRC-113 gene signature showed stronger prognostic ability than CRC stage: DFS (HR 3.5, 95% CI 1.7–7.5, *p* = 1.08e-03), OS (HR 2.9, 95% CI 1.5–5.7, *p* = 1.55e-03), and DSS (HR 5.0, 95% CI 1.9–13.2, *p* = 1.08e-03) in univariate analysis, and DFS (HR 3.1, 95% CI 1.6–6.3, *p* = 1.18e-03), OS (HR 2.9, 95% CI 1.5–5.6, *p* = 2.01e-03), DSS (HR 5.4, 95% CI 2.0–14.8, *p* = 1.10e-03) in multivariate analysis. No significant difference was obtained in other clinical variables.

**Table 1:** Clinicopathological features of CRC patients in two risk groups of GSE17538 discovery data set

Variables		Total	Low risk	High risk	*p* (χ^2^- test)
Number of patients (%)		145	72 (49.7)	73 (50.3)	
**Gender**	**Female**	69 (47.6)	32 (44.4)	37 (50.7)	0.507
	**Male**	76 (52.4)	40 (55.6)	36 (49.3)	
**Age**	**<70**	82 (56.6)	40 (55.6)	42 (57.5)	0.810
	**≥70**	63 (43.5)	32 (44.4)	31 (42.5)	
**AJCC stage**	**I**	24 (16.6)	17 (23.6)	7 (9.6)	8.45e-03
	** II**	55 (37.9)	29 (40.3)	26 (35.6)	
	** III**	56 (38.6)	19 (26.4)	37 (50.7)	
	** IV**	10 (6.9)	7 (9.7)	3 (4.1)	
**Grade**	**Well**	15 (10.3)	10 (13.9)	5 (6.8)	0.380
	**Moderately**	111 (76.6)	53 (73.6)	58 (79.5)	
	**Poorly**	19 (13.1)	9 (12.5)	10 (13.7)	
**Race**	**Black**	7 (4.8)	4 (5.6)	3 (4.1)	0.745
	**Caucasian**	122 (84.1)	60 (83.3)	62 (85.0)	
	**Hispanic**	1 (0.7)	0 (0)	1 (1.4)	
	**others**	15 (10.3)	8 (11.11)	7 (9.6)	
**DFS**	** 0**	109 (75.2)	63 (87.5)	46 (63.0)	6.43e-04
	** 1**	36 (24.8)	9 (12.5)	27 (37.0)	
**DSS**	** 0**	117 (80.7)	67 (93.1)	50 (68.5)	1.79e-04
	** 1**	28 (19.3)	5 (7.0)	23 (31.5)	
**OS**	** 0**	101 (69.7)	60 (83.3)	41 (56.2)	3.74e-04
	** 1**	44 (30.3)	12 (16.7)	32 (43.8)	

AJCC, American Joint Committee on Cancer; NA, not applicable; DFS, disease-free survival; DSS, disease specific survival; OS, overall survival; *p*-values were obtained from the χ^2^-test.

**Table 2:** Univariate and multivariate Cox proportional hazard regression analyses of clinical variables in discovery data set

Variable	DFS						OS						DSS					
	Univariate			Multivariate			Univariate			Multivariate			Univariate			Multivariate		
	HR	95% CI	*p* value	HR	95% CI	*p* value	HR	95% CI	*p* value	HR	95% CI	*p* value	HR	95% CI	*p* value	HR	95% CI	*p* value
**Age**	0.984	0.961–1.007	0.160	0.991	0.965–1.018	0.525	1.008	0.985–1.032	0.482	1.016	0.991–1.041	0.994	0.201	0.967–1.022	0.682	1.004	0.972–1.036	0.816
**Gender**	1.000	0.519–1.924	0.999	1.172	0.561–2.451	0.673	1.046	0.575–1.903	0.882	1.036	0.547–1.961	1.020	0.914	0.479–2.172	0.958	1.205	0.525–2.764	0.660
**Grade**	1.848	0.938–3.642	0.076	1.323	0.648–2.703	0.442	1.702	0.919–3.152	0.091	1.248	0.659–2.364	1.960	0.496	0.908–4.231	0.086	1.378	0.611–3.107	0.439
**AJCC stage**	2.047	1.351–3.100	7.20e-04	1.884	1.207–2.939	5.29e-03	1.819	1.251–2.645	1.82e-03	1.862	1.202–2.884	5.38e-03	2.084	1.288–3.372	2.77e-03	2.252	1.206–4.208	0.011
**Race**	0.891	0.313–2.539	0.829	1.036	0.355–3.028	0.948	1.710	0.526–5.561	0.372	0.569	0.304–1.066	1.603	0.078	0.377–6.809	0.523	1.950	0.451–8.442	0.371
**Risk**	3.525	1.656–7.501	1.08e-03	3.140	1.573–6.267	1.18e-03	2.921	1.504–5.671	1.55e-03	2.870	1.470–5.603	2.01e-03	5.018	1.907–13.201	1.08e-03	5.372	1.957–14.745	1.10e-03

AJCC, American Joint Committee on Cancer; DFS, disease-free survival; DSS, disease specific survival; OS, overall survival; HR, hazard ratio; CI, Confidence Interval; *p*-values were obtained from the χ^2^-test.

### Validation of CRC-113 gene signature in independent validation data sets

**Figure 2:**
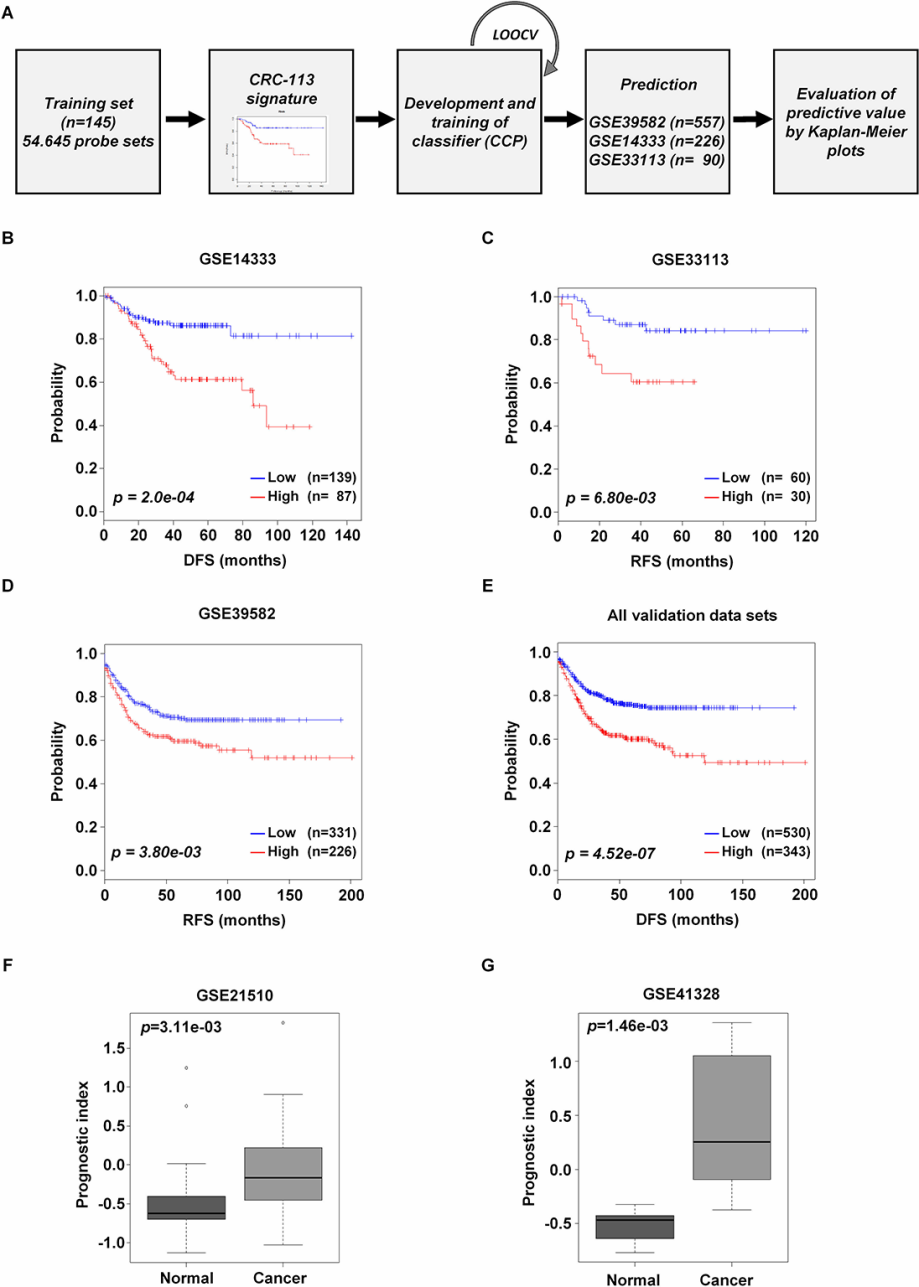
Prognostic significance of CRC-113 gene signature in independent validation data sets **A.** The flowchart of the strategy used for the generation of the risk prediction model and evaluation of risk outcome, based on CRC-113 gene signature. **B–D.** GSE39582, GSE14333, and GSE33113, **E.** all combined validation data sets were classified by CRC-113 gene signature into low and high risk, and evaluated by Kaplan-Meier analyses. **F** and **G.** GSE21510 and GSE41328 validation data sets with normal and cancer tissues. Box plots indicate prognostic differences in each group. In B-E, p values were computed by log-rank test. In F and G, p values were obtained by student t-test.

To evaluate the robustness of the CRC-113 classifier, we validated the CRC-113 gene signature in three independent data sets of colorectal cancer. The two risk groups were distinguished, based on their prognostic index of each patient (Supplementary Figure S2). A flow chart of the procedure used to validate the external data sets was provided (Figure 2A). During leave-one-out cross-validation (LOOCV), the specificity and the sensitivity for correctly predicting risk were 0.972 and 0.932 in CCP, respectively. The expression patterns of CRC-113 gene signature for each validation data set presented the low and high risk patient groups into two clusters (Supplementary Figure S3A–S3C). In the GSE14333 validation data set, CRC-113 gene signature distinguished 139 (61.5%) and 87 (38.5%) patients as the low and high risk groups, respectively. The Dukes' stage and the DFS were significantly correlated to our classification (*p* = 2.98e-59 and *p* = 4.57e-09, respectively, Supplementary Table S2). In the GSE33113 validation data set, 66 (66.7%) and 30 (33.3%) patients were predicted as low and high risk groups, respectively. Recurrence-free survival (RFS) was significantly correlated to our classification (*p* = 0.011, Supplementary Table S3). In the GSE39582 validation data set, 331 (59.4%) and 228 (40.6%) patients were classified into low and high risk groups, respectively. AJCC stage and relapse-free survival (RFS) were significantly correlated to our classification (*p* = 0.034 and *p* = 3.00e-03, respectively, Supplementary Table S4). CRC-113 gene signature significantly classified patients into low and high risk groups in three independent validation data sets on both univariate and multivariate analyses (Supplementary Table S5–S7). In multivariate analyses, CRC-113 gene signature showed prognostic significance for risk in these three different validation data sets: DFS of GSE14333 (HR 2.2, *p* = 9.27e-03, Supplementary Table S5), RFS of GSE33113 (HR 3.2, *p* = 0.014, Supplementary Table S6) and RFS of GSE39582 (HR 1.7, *p* = 8.37e-04, Supplementary Table S7). Kaplan-Meier plots indicated significant differences in these three validation data sets: GSE14333 (*p* = 2.0e-04, Figure 2B), GSE33113 (*p* = 6.80e-03, Figure 2C) and GSE39582 (*p* = 3.80e-03, Figure 2D). The combined validation data sets were also significantly classified into low and high risk groups (*p* = 4.52e-07, Figure 2E).

### Validation of CRC-113 gene signature in colorectal cancer and normal tissue

To further evaluate whether there was a significant difference between CRC tissues and normal tissues based on gene expression of CRC-113 gene signature, we analyzed two independent data sets, GSE21510 [26] and GSE41328 [27], for which there was no available survival information on public database. As shown in results, the prognostic indices of normal subjects were evidently lower than those of colorectal cancer patients in GSE21510 (*p* = 3.11e-03, Figure 2F) and GSE41328 (*p* = 1.46e-03, Figure 2G) data sets, respectively. None of the tissue samples from non-colorectal cancer patients were predicted as indicating high risk.

### Validation of CRC-113 gene signature in stage II and III CRC patients

**Figure 3:**
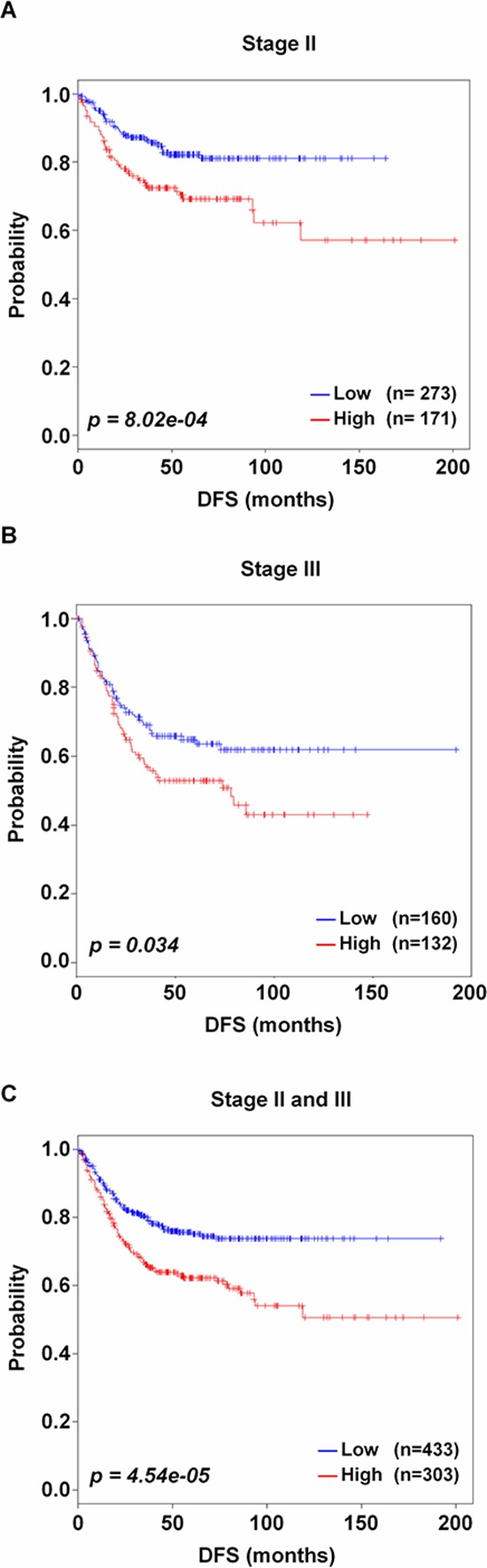
Kaplan-Meier plots of stage II and/or III patients Incorporation of CRC-113 gene signature into patients with **A–C.** stage II, stage III, and stage II and III, respectively in combined validation data sets. Each group was classified by CRC-113 gene signature into low and high risk, and evaluated by Kaplan-Meir analyses. *p* values were computed by log-rank test.

CRC patients in stage II and III frequently develop recurrence after treatment, while patients in stage I are usually cured by surgery alone [5]. Thus, we investigated whether CRC-113 gene signature could suitably classify patients with stage II and/or III into two risk groups in discovery and/or validation data sets. The discovery data set included patients with survival information in stage II (*n* = 55) and III (*n* = 56), and the validation data sets comprise patients with stage II (*n* = 444) or III (*n* = 292). All patients were labeled according to Dukes' classification system in the GSE14333 validation data set. Thus, we categorized patients with Dukes' B and C stages to AJCC stage II and III, respectively. In all data sets, stage II patients showed a good outcome, whereas stage III patients had a relative poor outcome (80.3% and 63.5% in 5-year DFS, respectively). As expected, CRC-113 gene signature significantly stratified the stage II and/or III patients into low and high risk groups (Figure 3). The patients with high risk (*n* = 303, 41.2%) showed poorer outcomes than those with low risk (*n* = 433, 59.8%) in stage II and/or III (*p* = 8.02e-04 for stage II, *p* = 0.034 for stage III and *p* = 4.54e-05 for stage II and III, respectively, Figure 3A–3C). Additionally, with the discovery data set, we also observed similar results in stage II (*p* = 1.38e-04), stage III (*p* = 0.012), and stage II and III (*p* = 1.13e-06) (Supplementary Figure S4A–S4C). This CRC-113 gene signature could clearly classify patients in stage I with or without patients from the discovery data set (*p* = 0.033 and *p* = 7.82e-03, respectively, Supplementary Figure S4D and S4E), but not in stage IV patients even including patients from discovery data set (Supplementary Figure S4F).

### Association of CRC-113 gene signature with molecular pathways and mutations

**Figure 4:**
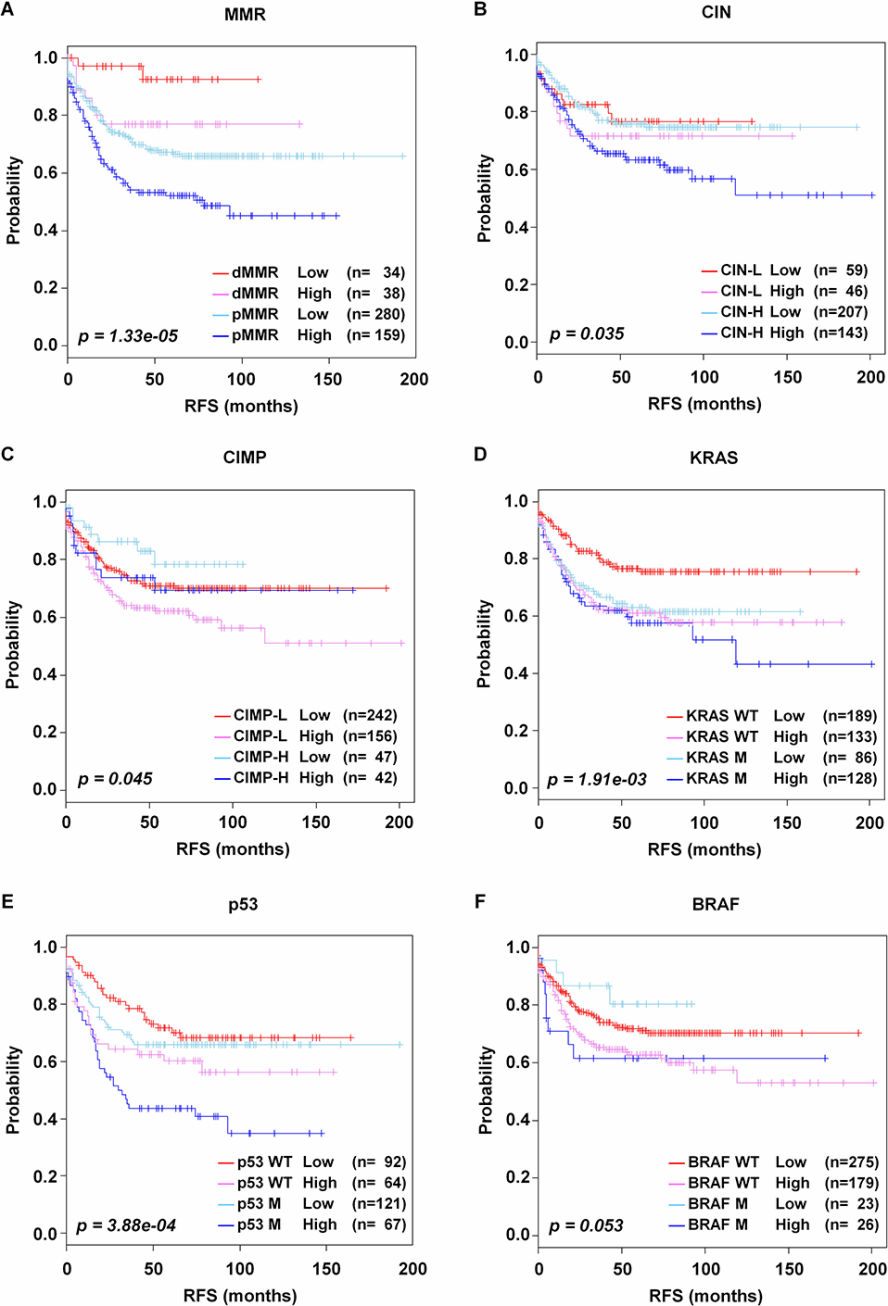
Kaplan-Meier survival analysis of CRC-113 gene signature with molecular pathways and gene mutations Incorporation of CRC-113 gene signature into **A–F.** MMR, CIMP, CIN, KRAS, BRAF, and p53 status of CRC patients. Each group was classified by CRC-113 gene signature into low and high risk, and evaluated by Kaplan-Meir analyses. *p*-values were obtained from the χ2-test.

Traditional CRC development involves stepwise accumulation of genetic alterations [28], which is substantially more complex than that originally envisioned with three distinct pathways of genetic instability: MMR, CIMP and CIN [29]. The MMR dysfunction causes MSI which is the condition of genetic hypermutability. The CIMP inactivates tumor suppressor genes via genome hypermethylation. The CIMP is also relevant to BRAF mutation [30]. The CIN phenotype results from the accumulation of numerical or structural chromosomal abnormalities (aneuploidy) [31], and is strongly related to KRAS and p53 mutations [32]. However, it still remains undefined to evaluate the heterogeneity of CRC. Based on the information of these genetic and epigenetic alterations presented in the GSE39582 data set, MMR and KRAS could classify the patients into low and high risk groups (*p* = 9.26e-04 and *p* = 0.023, respectively, Supplementary Figure S5A and S6A). However, the other DNA alterations did not contribute to stratification of patients into two prognostic risk groups (CIN and CIMP, Supplementary Figure S5D and S5G; p53 and BRAF, Supplementary Figure S6D and S6G). We thus investigated whether the CRC-113 gene signature could further stratify the CRC patients associated with the molecular subtypes. We first incorporated CRC-113 gene signature with each of these DNA alteration factors. In association analysis using χ^2^ test, the CRC-113 gene signature risk was remarkably interrelated with each DNA alteration: MMR (*p* = 1.33e-05, Figure 4A), CIN (*p* = 0.035, Figure 4B), CIMP (*p* = 0.045, Figure 4C), KRAS (*p* = 1.91e-03, Figure 4D) and p53 status (*p* = 3.88e-04, Figure 4E), except for BRAF status (*p* = 0.053, Figure 4F). The high risk patients with pMMR showed the highest risk outcome among the four sub-groups. The high risk patients with KRAS wild type (WT) presented similar poor-prognostic outcomes compared to the patients with KRAS mutant (M). Moreover, CRC-113 gene signature significantly exhibited further hierarchical discrimination in the status of DNA alterations: MMR proficient (pMMR, *p* = 6.34e-04) and MMR deficient (dMMR, *p* = 0.05) (Supplementary Figure S5B and S5C); CIN-high (*p* = 5.24e-03) and CIMP-low (*p* = 0.032) (Supplementary Figure S5F and S5H), KRAS WT (*p* = 1.32e-03, Supplementary Figure S6B), p53 M (*p* = 1.05e-03, Supplementary Figure S6F), and BRAF WT (*p* = 0.024, Supplementary Figure S6H). Patients with pMMR frequently presented CIMP-low and CIN-high phenotypes (78.4%, *n* = 269 of 343), whereas there was no significant relationship in dMMR, CIMP and CIN. Additionally, KRAS, BRAF and p53 mutations did not show any interrelationship. The association between each DNA alteration and CRC-113 gene signature was summarized in Supplementary Table S8.

### Association of CRC-113 gene signature with advantage of adjuvant chemotherapy

**Figure 5:**
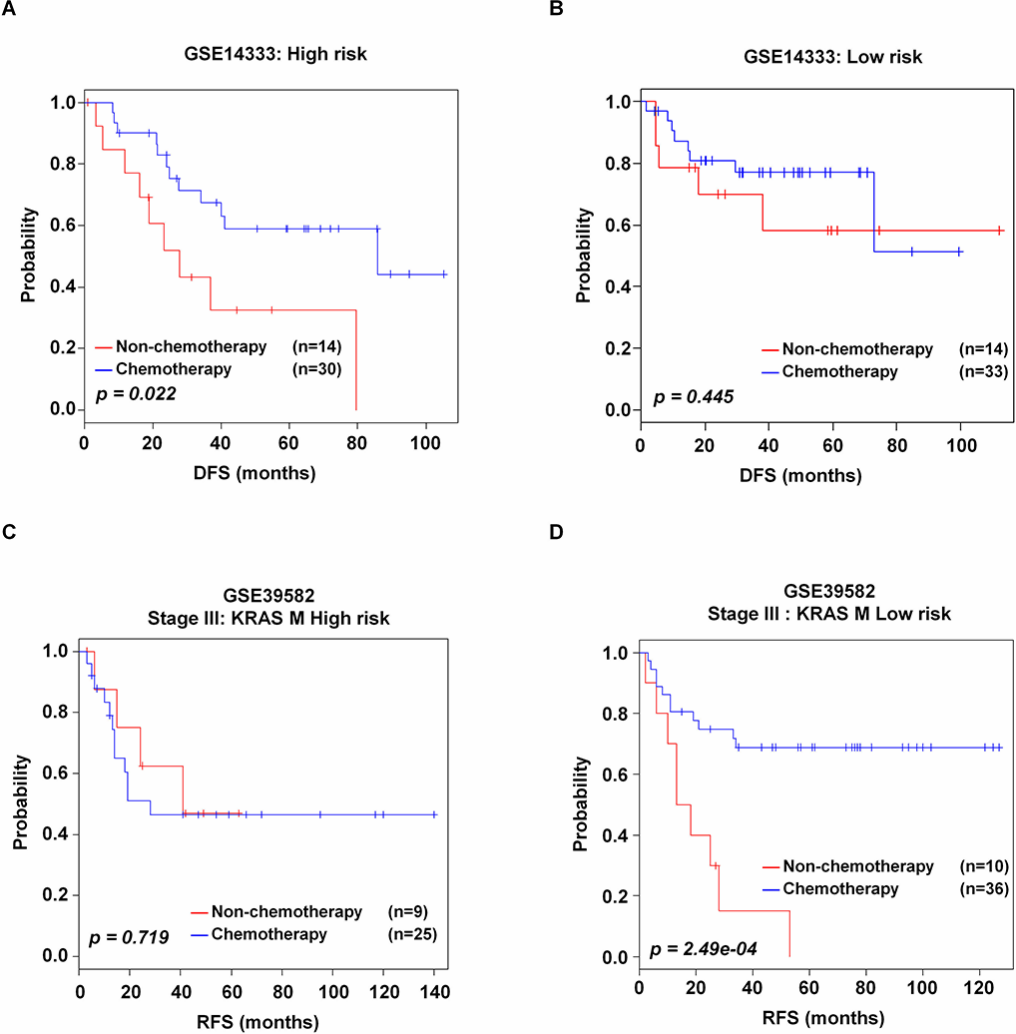
Kaplan-Meier survival analysis of stage III CRC with adjuvant chemotherapy **A** and **B.** high risk and low risk groups in GSE14333. **C–D.** high risk and low risk groups of KRAS M in GSE39582. Patients were separated according to chemotherapy treatment, and the chemotherapeutical advantage was evaluated by Kaplan-Meir analyses. *p* values were computed by log-rank test.

Adjuvant chemotherapy for stage III CRC has been shown to improve survival rate, and is currently recommended as standard therapy [33, 34]. Thus, in order to examine the association of the signature with response to adjuvant chemotherapy, we performed subgroup analysis with patients in stage III of GSE14333 and GSE39582. In the GSE14333 validation data set, the patients in stage C received standard adjuvant chemotherapy (either single agent 5-fluouracil/capecitabine or 5-fluouracil and oxaliplatin). In the GSE39582 validation data set, the stage III patients received standard adjuvant chemotherapy with 5-fluorouracil and leucovorin. Chemotherapy itself showed therapeutic benefit for DFS in the GSE14333 validation data set (*p* = 0.037, Supplementary Figure S7A), but did not give advantage for recurrence in the GSE39582 validation data set (*p* = 0.554, Supplementary Figure S7B). By incorporating CRC-113 gene signature into chemotherapy information, the high risk patients with stage III of GSE14333 validation data set were shown to obtain the benefit compared to patients without adjuvant chemotherapy (*p* = 0.022, Figure 5A). In contrast, low risk patients with stage III of the GSE14333 validation data set did not have significant difference in chemotherapy treatment (*p* = 0.445, Figure 5B). Interestingly, the stage III patients in both low and high risk groups of GSE39582 validation data set did not benefit from chemotherapy (Supplementary Figure S7C and S7D). We also applied CRC-113 gene signature to other stages; however, all the patients of these stages did not achieve benefit with chemotherapy treatment (data not shown). Additionally, we investigated whether incorporation of CRC-113 gene signature into DNA alterations could give chemotherapeutical benefit to stage III patients of the GSE39582 validation data set. Without incorporation of CRC-113 gene signature, only the patients with KRAS M among stage III had chemotherapeutical benefit (*p* = 0.018, Supplementary Figure S7E). However, with incorporation of the CRC-113 gene signature, high risk patients had no benefit in adjuvant chemotherapy (*p* = 0.719, Figure 5C), whereas low risk patients receiving adjuvant chemotherapy showed better prognosis (*p* = 2.49e-04, Figure 5D).

### Incorporation of CRC-113 gene signature into a published molecular subtype classifier

**Figure 6:**
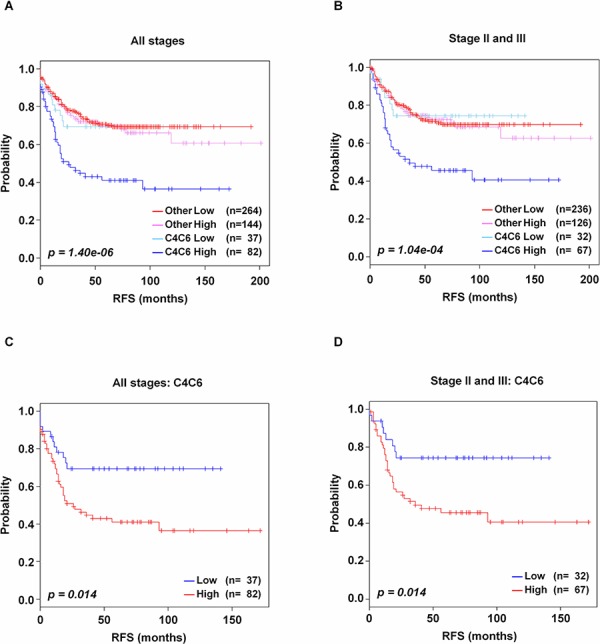
Kaplan-Meier survival analysis of CRC-113 gene signature with subgroups of the GSE39582 validation data set Incorporation of CRC-113 gene signature into patients with **A.** all stage, **B.** stage II and III, **C.** stage II, and **D.** stage III. Each group was classified by CRC-113 gene signature into low and high risk, and evaluated by Kaplan-Meir analyses. *p*-values were obtained from the χ2-test.

Marisa, *et al* (GSE39582 validation data set) [23] previously suggested six molecular subtypes for the predicting prognosis of CRC recurrence. These subtypes were associated with distinct clinicopathological characteristics, molecular mutations, gene expression signature and signaling pathways. The six subtypes were termed according to their biological characteristics as follows: C1 (CIN_ImmuneDown_), C2 (dMMR), C3 (KRASm: KRAS-mutant), C4 (CSC: cancer stem cell), C5 (CIN_WntUp_) and C6 (CIN_normL_ The subtypes were finally categorized by two distinct groups: a poor-prognosis group (‘C4C6′: C4 and C6), and all other subtypes as the good-prognosis (‘Others’: C1, C2, C3 and C5), corresponding with the prognostic difference. They reported that the C4 and C6 subtypes were enriched for stem cell-like signature from both of a mouse intestinal stem cell signature [35] and a human colon top and bottom crypt signature [36], and a normal-like signature from a breast cancer signature [37], respectively. Especially, 1108 probe sets were used for subtype-discrimination, which shared 53 probe sets with CRC-113 gene signature (Supplementary Table S1). Thus, we investigated the association between CRC-113 gene signature and these two distinct prognostic groups. After incorporation of CRC-113 gene signature, our signature risk was significantly associated with the binary classification in all stages (*p* = 1.40e-06, χ^2^-test, Figure 6A), and a similar result was found in the analysis of the stage II and III combined group (*p* = 1.40e-04, χ^2^-test, Figure 6B). Here, we identified the poorest prognostic sub-group, C4C6-high, from the ‘C4C6’ group. Our classifier further stratified this ‘C4C6’ group into low and high risk groups in all stages (*p* = 0.014, Figure 6C), stage II and III (*p* = 0.014, Figure 6D), while the ‘Others’ group was not further classified (data not shown). Among the patients of ‘C4′ (*n* = 59) and ‘C6’ (*n* = 60), 58 and 24 patients (*n* = 82, 68.9%), respectively, belonged to high risk patients of CRC-113 gene signature.

### Gene ontology term enrichment analysis and visualization of CRC-113 gene signature

To identify the biological function of the genes in the CRC-113 gene signature, we performed GO enrichment analysis in DAVID, and then identified 42 significant GO terms (biological process), including biological adhesion, cell adhesion, cell motility, extracellular matrix organization and response to wounding. The false discovery rates (FDRs) were estimated using the procedure of Benjamini (*p* < 0.05, Supplementary Table S9). GO term redundancy was removed and visualized in the semantic space via REViGO, representing functional clusters (Supplementary Figure S8).

### Protein network in CRC-113 gene signature

To verify potential protein interactions of 77 genes in the CRC-113 gene signature, we generated a molecular network by introducing these 77 genes into STRING, a molecular tool that was able to elaborate physical and functional associations among proteins. As shown in results, 69 out of 77 genes were closely connected in a single network (Supplementary Figure S9).

## DISCUSSION

In colorectal cancer, accurate prognostic prediction for recurrence and mortality after surgery is frequently limited due to molecular heterogeneity. Thus, it is necessary to correctly identify individual recurrence risk and adjuvant chemotherapeutical benefit. Several studies have previously shown that the gene signatures are capable of prognosticating in CRC patients, but no gene signature has been clinically useful yet. To address this issue, we established a CRC-113 gene signature which could be valuable to predict disease recurrence and adjuvant chemotherapy effect by using a large patient sample size with a long follow-up time and the same platform. We applied the supervised method and avoided model overfitting by LOOCV. The robustness of the CRC-113 gene signature was supported by the high sensitivity (0.972) and specificity (0.932) values, and the reproducibility through significant association between the predicted outcome and patient prognosis in validation data sets. Independence of CRC-113 gene signature as a prognostic marker was reinforced by the results using various approaches. By incorporating DNA alterations, we found that the CRC-113 gene signature which could further stratify patients into prognostic subdivisions has important clinical value to help guide judicious treatment decisions. Additionally, subgroup analysis of patients with stage III cancer only indicated that CRC-113 gene signature might predict which patients would benefit from adjuvant chemotherapy for DFS. Finally, we demonstrated that CRC-113 provides a new insight to elucidate CRC heterogeneity.

The current AJCC pathological staging criteria cannot accurately predict patient survival. Approximately 25% of CRC patients present metastatic features, and pathological staging fails to correctly predict recurrence in many patients undergoing curative surgery for CRC due to the heterogeneity [38, 39]. We evidently showed that the CRC-113 gene signature supplies the lack of pathological staging via further stratification of patients into significant low and high risk groups in each CRC stage. Unfortunately, CRC-113 gene signature was not correlated to demographic disparities such as age, gender and ethnicity for prognosis prediction. Actually, these factors have been considered to be the risk factors in CRC [40, 41]. For example, incidence or mortality rates of CRC are statistically highest in black men and women, followed by white, Hispanic, Asian/Pacific Islander, and American Indian/Alaska Native people [41]. At present, it remains to be answered why our gene signature does not reflect the demographic disparities.

In colorectal carcinogenesis, there are three distinct genetic pathways: MSI, CIMP and CIN [29]. However, the patients evaluated by these molecular markers still differ remarkably in prognosis and therapeutic responses [42]. We found that CRC-113 gene signature further stratified patients in combination with MMR status; high risk patients with pMMR presented the poorest prognosis. It also stratified CIMP-Low or CIN-High patients. Moreover, CRC-113 gene signature further stratified the patients with KRAS and BRAF WTs, and p53 M. Therefore, we argue that CRC-113 gene signature can overcome limitations of the conventional molecular markers via further stratification in CRC predicting prognosis. Interestingly, the number of the high risk group (*n* = 128, 59.8%) was relatively larger than that of the low risk group (*n* = 86, 40.2%) in patients with KRAS M, while the ratio was inclinable to be opposite in patients with KRAS WT (*n* = 133, 41.3% for high risk group; *n* = 198, 58.7% for low risk group). This result seemed reasonable for the relationship between KRAS status and risk classification, although the sample size was not sufficient to firmly conclude this observation. Meanwhile, poor-prognostic outcomes were shown in both groups of low risk with KRAS M and high risk with KRAS WT.

The identification of individual patients in need of optimized adjuvant therapy still remains as a major clinical concern. In both internal and combined-validation data sets, the CRC-113 gene signature clearly stratified stage III CRC patients into low and high risk groups. Subgroup analysis of patients with available data revealed that adjuvant chemotherapy improved DFS in high risk patients with stage III. Although CRC-113 gene signature could not predict delayed relapse after adjuvant chemotherapy, when combined with KRAS M, it helped to define stage III patients with delayed relapse. Our signature also showed that a subgroup of patients with low risk and KRAS M were more sensitive to chemotherapy. Additionally, we found that a subgroup of patients with high risk and KRAS M did not present adjuvant chemotherapeutical advantage. The CRC-113 gene signature might imply the potential benefit of adjuvant chemotherapy in patients with stage III CRC, although we agree that it would not be enough to make a strong conclusion for the predictive power due to the small number of patients used in these analyses.

In the comparative analysis between our gene signature and the six molecular subtypes (C1-C6) in the recent study of Marisa, et al [23], the C4 and C6 patients (‘C4C6’) were classified as a poorer-outcome group than the other group patients. The ‘C4C6’ subtypes presented down-regulation of cell growth and death pathways, and up-regulation of the epithelial–mesenchymal transition pathway. Our CRC-113 gene signature further stratified ‘C4C6’ patients into low and high risk groups, of which high risk patients in ‘C4C6’ belonged to the poorest subtype group. Especially, all of the C4 patients, except for one, were evaluated as high risk patients by CRC-113 gene signature. Interestingly, 36 genes in CRC-113 gene signature were overlapped with their subtype-discrimination probe sets that they reported [23].

The majority of genes in CRC-113 gene signature have critical roles in cell proliferation, angiogenesis, migration, invasion and metastasis of CRC. These genes include APOE [43], Bcat1 [44], CAV2 [45], COL1A1 [46], COL3A1 [47], COL5A2 and COL11A1 [48], COL10A1 [49], CTGF [50], FN1 [51, 52], HOPX [53], HOXC6 [54], LOX [55], NRP-1 [56], SERPINE1 [57], THBS2 [58], TM4SF1 [59], Versican [60], WIST1 [61] and WNT5A [62]. Also, CRC-113 gene signature includes a number of hypoxia and inflammation-related genes in various cancer such as AKAP12 [63], ANXA1 [64, 65], CCL11 [66], CTGF [67], FABP4 [68], FN1 [69], IGFBP3 [70], LOX [71], NOX4 [72, 73], NRP1 [74], OLR1 [75], SLC2A3 [76] and WNT5A [77], indicating that hypoxia and inflammation, which are two inseparable hallmarks in tumorigenesis [78], really play important roles in CRC pathogenesis. In addition, CRC-113 gene signature also contains epigenetics-related genes, which have pivotal roles in cancer development. NNMT controls hypomethylation of histones and other cancer-related proteins [79]. The hypermethylation of FBN1 and SFRP2 was reported as sensitive molecular markers for detecting CRC [80, 81]. Finally, many novel genes such as C5AR1, KRT80, FRMD6, OLFML2B, PRRX1 and ZNF532 are included, suggesting that CRC-113 gene signature contains new promising biomarkers for CRC diagnosis and potential therapeutic targets.

Conclusively, we developed a robust gene signature that is highly discriminative. We demonstrated that CRC-113 gene signature predicts individual patients at high risk of recurrence and mortality by integrating CRC heterogeneity. The prognostic value of our signature was statistically significant in the overall data sets, independently of the pathological staging. When incorporated into a clinical context and molecular subtypes, CRC-113 gene signature further stratified patients into two distinct prognostic risk groups to overcome the limitation of the conventional classification and molecular markers. Hence, we propose that our CRC-113 gene signature provides a basis for the rational design of potentially targetable markers for CRC prognostic prediction.

## MATERIALS AND METHODS

### Patient and gene expression data

All clinical and gene expression data are available on Gene Expression Omnibus database (http://www.ncbi.nlm.nih.gov/geo/) fulfilling the following criteria: a similar chip platform (Affymetrix U133 Plus 2.0 chips) with raw data CEL files (Table 3) and clinical information of patients on survival event and time (Table 4). The raw data were normalized using a robust multiarray averaging method [82, 83]. The 1,358 unique patients of six different CRC data sets were used in the analysis. Gene expression data of 340 patients who had no clinicopathological information were excluded from survival analysis. GSE17538 (*n* = 145, Moffitt Cancer Center, Vanderbilt Medical Center) was used as a discovery data set [17]. The validation sets were GSE14333 (*n* = 226, Royal Melbourne Hospital) [21], GSE33113 (*n* = 96, Academic Medical Center in Amsterdam) which included only stage II patients of CRC [22, 25] and GSE39582 (*n* = 557, the French Ligue Nationale Contre le Cancer) [23]. GSE21510 (*n* = 148, Tokyo Medical and Dental University Hospital) [26] and GSE41328 (*n* = 20, University of Illinois) [27], were used for comparing between normal subjects and CRC patients.

**Table 3:** CRC microarray data sets

GEO Number	Origin/Year	Chip type	References
GSE17538	USA, 2009	Affymetrix HG-U133_Plus_2	Smith, et al [17, 18]
GSE14333	Australia, 2010	Affymetrix HG-U133_Plus_2	Jorissen, et al [21]
GSE33113	Netherlands, 2011	Affymetrix HG-U133_Plus_2	de Sousa, et al [22, 25]
GSE39582	France, 2013	Affymetrix HG-U133_Plus_2	Marisa, et al [23]
GSE21510	Japan, 2011	Affymetrix HG-U133_Plus_2	Tsukamoto, et al [26]
GSE41328	USA, 2006	Affymetrix HG-U133_Plus_2	Lin, et al [27]

**Table 4:** Clinical characteristics of patients in discovery and validation data sets

Characteristics	Discovery data set	Validation data sets		
	GSE17538	GSE14333	GSE33113	GSE39582
Number of patients (Patients used)	238 (145)	290 (226)	96 (90)	566 (557)
Median age (years)	65	67	73.98	68.1
Gender (male/female)	124/114	164/126	42/48	310/256
AJCC stage				
0	0	−	0	4
I	28	−	0	33
II	72	−	96	264
III	76	−	0	205
IV	56	−	0	60
N/A	6	−	0	0
Dukes' stage				
A	−	44	−	−
B	−	94	−	−
C	−	91	−	−
D	−	61	−	−
N/A	−	0	−	−
Chemotherapy				
Yes	−	117	−	233
No	−	172	−	316
N/A	−	1	−	17
DFS	145 (28.36)^1^	226 (39.32)^1^	−	−
OS	177 (41.49)^1^	−	−	−
RFS*	−	−	90 (39.32)^1^	557 (43.00)^1^
DSS	232 (41.52)^1^	−	−	−

1 presents median months of followup times; AJCC, American Joint Committee on Cancer; NA, not applicable; OS, overall survival; DFS, disease-free survival; RFS*, recurrence-free survival for GSE33113 validation data set, relapse-free survival for GSE39582 validation data set; DSS, disease specific survival.

### Development of the prognostic gene expression signature

A gene expression signature to predict prognostic risk was developed from the GSE17538 discovery data set. Gene expression and disease-free survival (DFS) data were combined to build a gene expression profiling-based survival classifier. The 54,675 probe sets were filtered by at least 2 absolute value of log2 scale which represented the same gene expression level. The univariate Cox proportional hazard regression (*p* < 0.001) was then used to identify the DFS-associated gene expression signature from the discovery data set. Regarding predicting prognosis, probes from the survival signature were applied to the survival risk prediction analysis [84]. This method used the principal component from the discovery data set and produced a prognostic index for each patient. The prognostic index was computed by the formula ∑_i_w_i_ x_i_ - 0.256901 where w_i_ and x_i_ were the weight and logged gene expression for the i-th gene, respectively. We attempted to divide the patients into two groups based on a median prognostic index of −0.04444. Patients were assigned to the high risk group if their prognostic indices were greater than the median value, whereas the low risk group was composed of patients with the prognostic indices that were equivalent to or less than the median value.

### Validation of the prognostic signature

The validation of the gene signature was accomplished on independent data sets. Gene expression data from different data sets were adjusted individually by subtracting the median expression value across the samples. To further refine this model and to sub-stratify the predicted outcomes, Compound Covariate Predictor (CCP) was utilized as a class prediction algorithm [85]. The robustness was estimated by the misclassification rate that was determined during the leave-one-out cross-validation (LOOCV) in the training set.

The Kaplan-Meier survival analyses were performed after the patient classification into two risk groups, and Chi-square (χ^2^) and log-rank tests were used to evaluate the survival risk between two predicted subgroups of patients. The univariate and multivariate Cox proportional hazard regression analyses were used to evaluate independent prognostic factors associated with survival, and then gene signature, tumor grade and pathological characteristics were employed as covariates.

### Pathway analysis

Gene ontology (GO) biological process enrichment analysis was carried out using the Database for Annotation, Visualization and Integrated Discovery (DAVID) bioinformatics tool (http://david.abcc.ncifcrf.gov/) [86]. The results of the GO analysis were visualized in semantic similarity-based scatterplots via REViGO [87], a web server that summarized GO terms by removing redundant ones. The allowed similarity was chosen to be small (0.5), and the semantic similarity measure was ‘SimRel’.

### STRING analysis

Protein-protein interactions were predicted using the Search Tool for the Retrieval of Interacting Genes/Proteins (STRING) database v10.0 (http://www.string-db.org/). Proteins were linked based on the following six criteria; neighborhood, gene fusion, co-occurrence, co-expression, experimental evidence and existing databases [88].

### Statistical methods of microarray data

Microarray data and heatmap were analyzed using BRB-Array Tools Version 3.0 (http://linus.nci.nih.gov/BRB-ArrayTools.html) [89]. All other statistical analyses were accomplished in the R language environment (http:///www.r-project.org) and Statistical Package for Social Sciences (SPSS) software (version 20, SPSS Inc, Chicago, IL, USA). In all statistical analyses, *p* value of less than 0.05 was considered significant.

## SUPPLEMENTARY FIGURES AND TABLES


