# Supplementary material for: CRC-113 gene expression signature for predicting prognosis in patients with colorectal cancer
Source: Oncotarget. 2015 Sep 11;6(31):31674–92. doi: 10.18632/oncotarget.5183 (PMC4741632; doi:10.18632/oncotarget.5183)
Supplement: Supplementary file 1 [file oncotarget-06-31674-s001.pdf]

SUPPLEMENTARY FIGURES AND TABLES

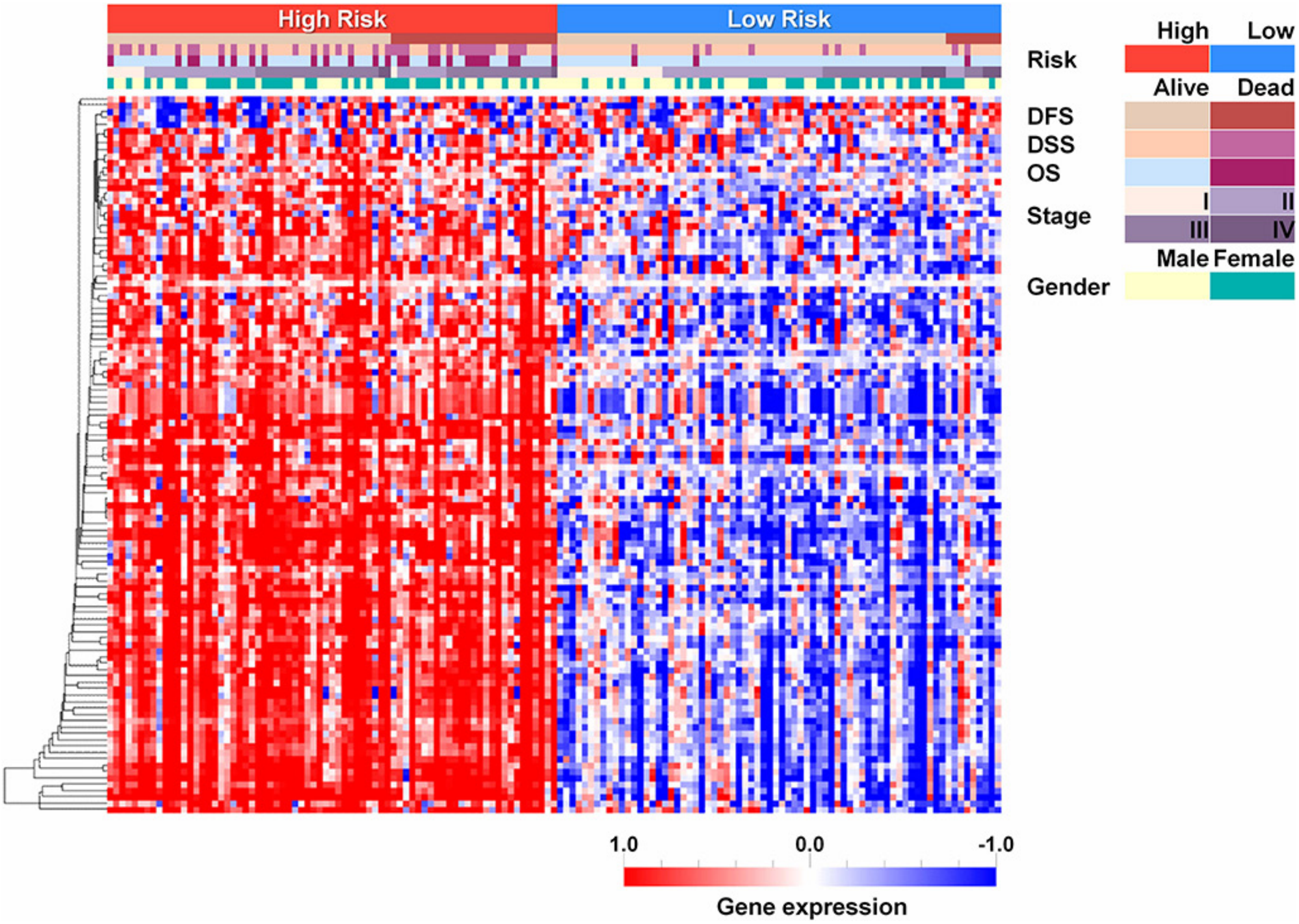

Supplementary Figure S1: Hierarchical cluster analysis of CRC-113 gene signature in the discovery data set. Survival and clinical information were associated with the heatmap of the two risk groups in the discovery data set.

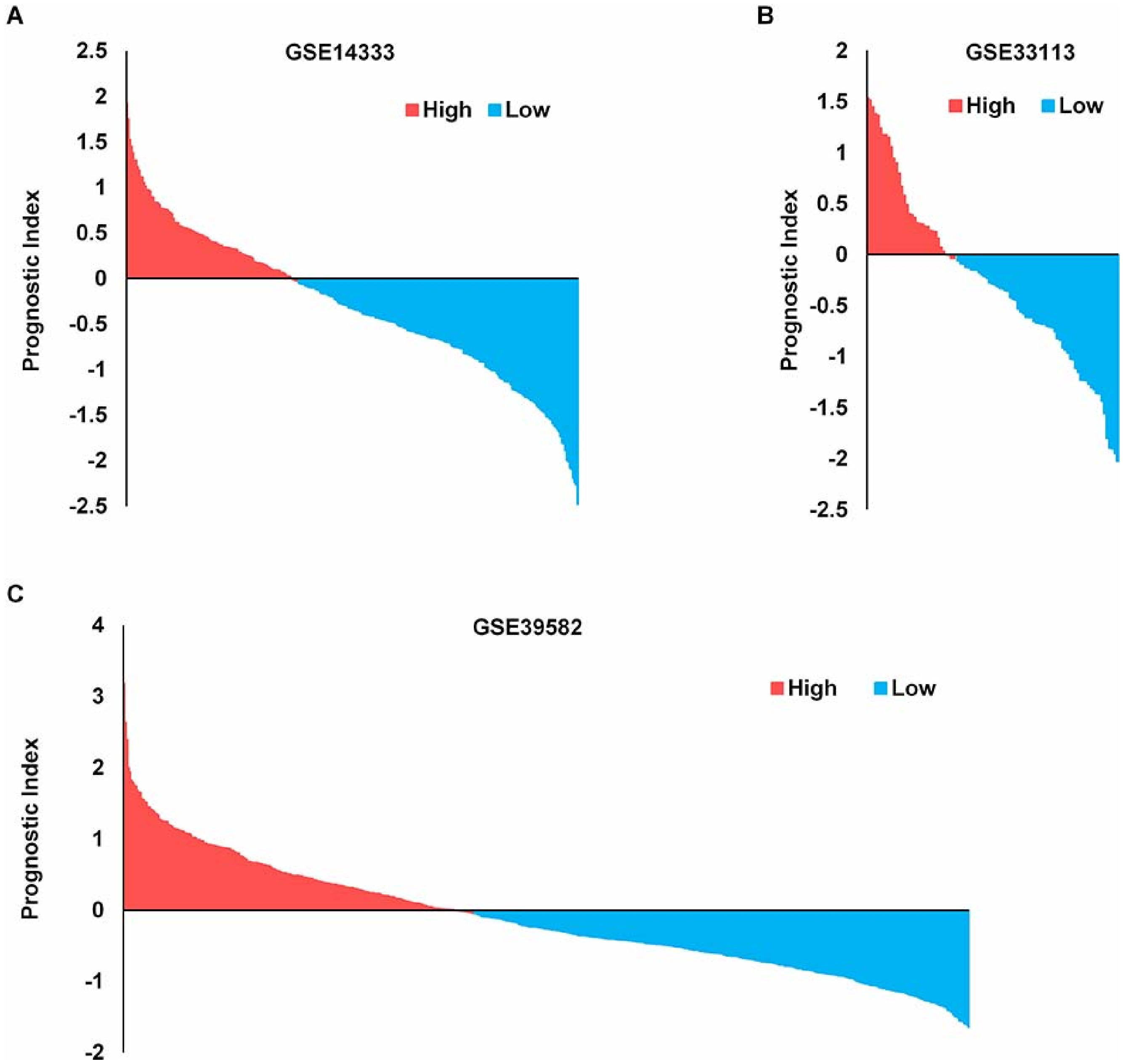

Supplementary Figure S2: Prognostic indices of CRC-113 gene signature in validation data sets. The relative prognostic indices were based on CRC-113 gene signature expression of each patient in **A–C.** GSE14333, GSE39583 and GSE33113 validation data sets.

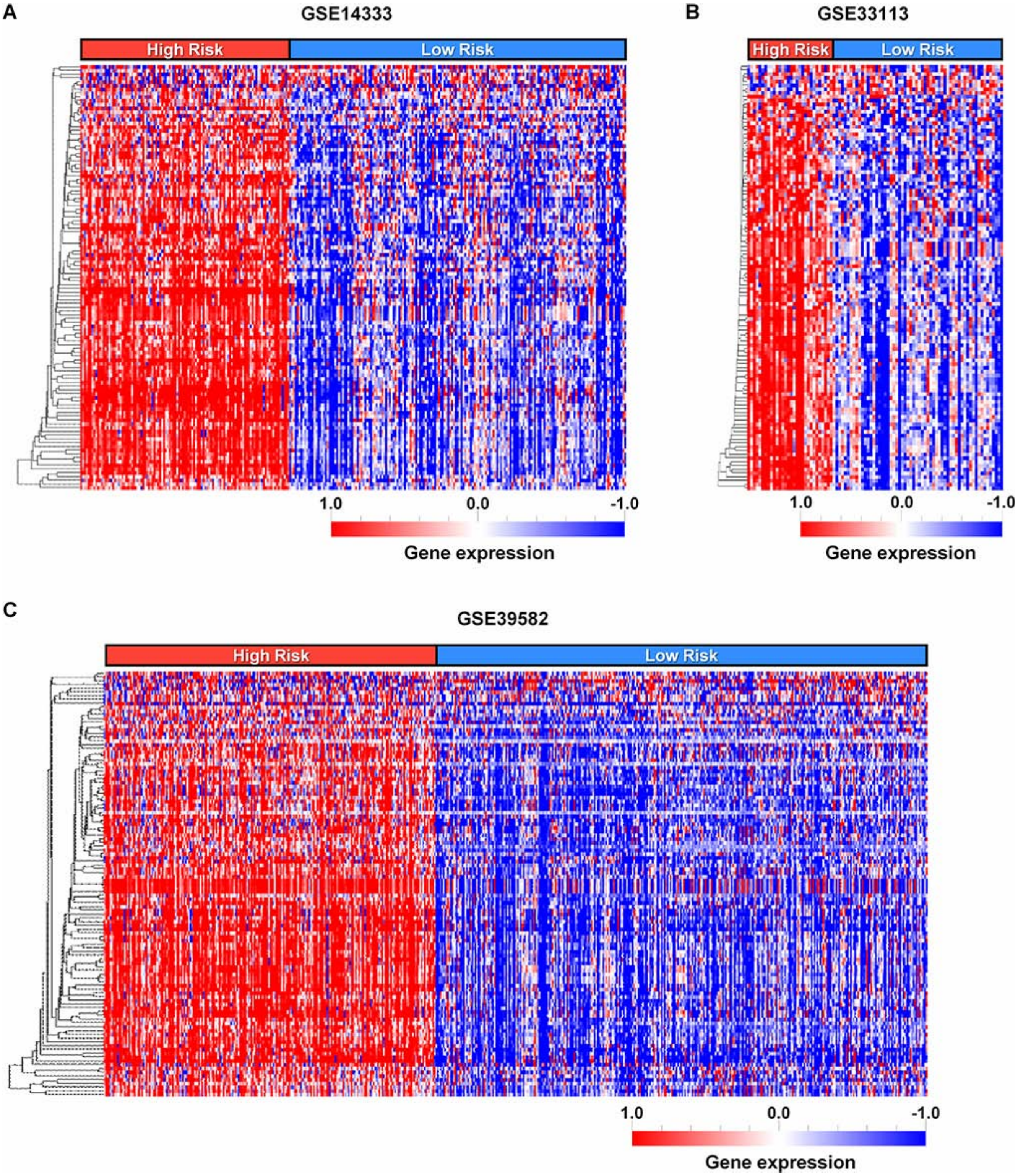

Supplementary Figure S3: Hierarchical cluster analysis of CRC-113 gene signature in the validation data sets. **A–C.** GSE14333, GSE39583 and GSE33113 validation data sets. The scale bar shows color-coded differential expression, with red indicating higher expression and blue lower expression.

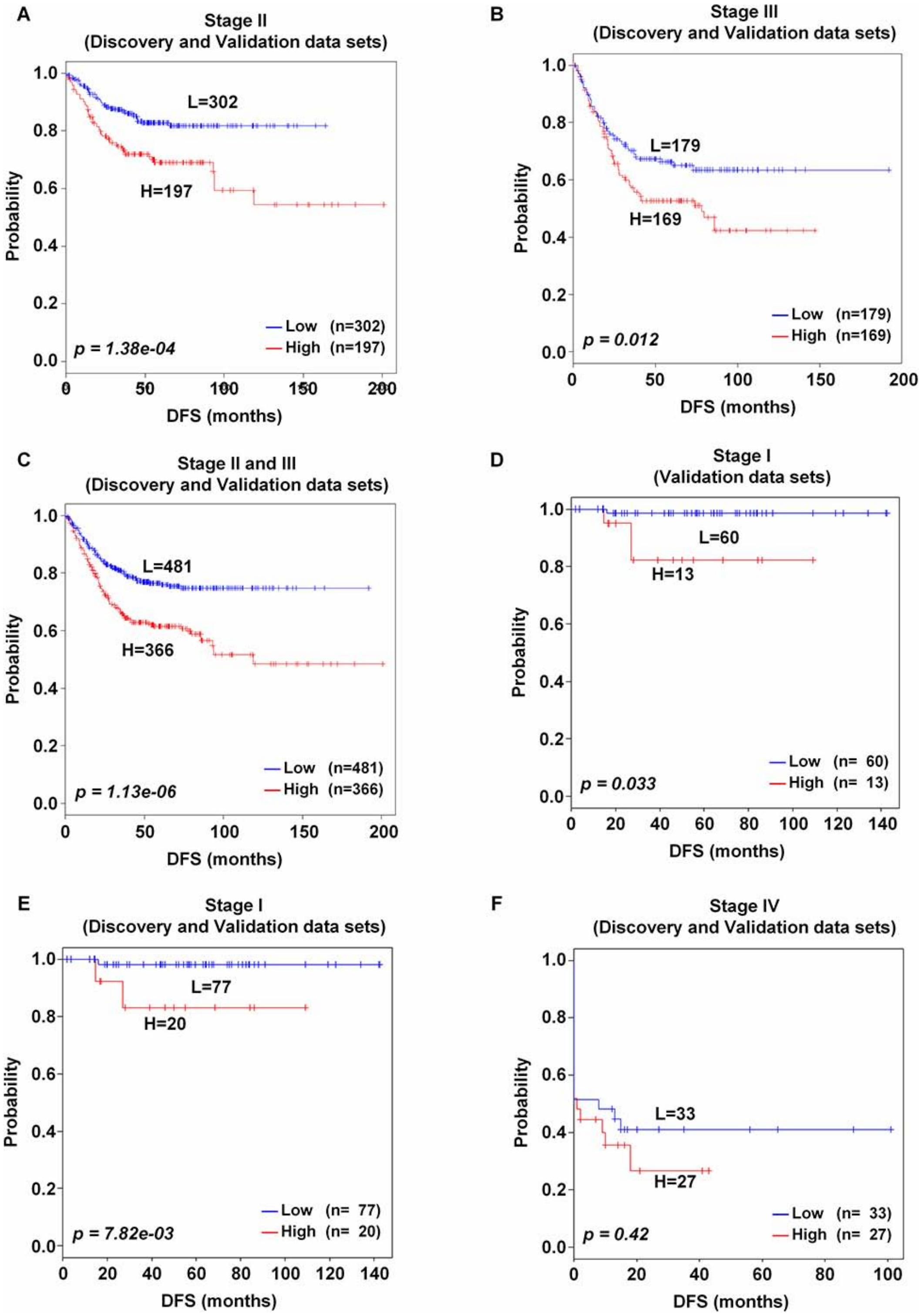

Supplementary Figure S4: Kaplan-Meier survival analysis of stage II and III CRC in the discovery and validation data sets. A. Stage II patients in the combined discovery and validation data sets. **B.** Stage III patients in the combined discovery and validation data sets. **C.** Stage II and III patients in the combined discovery and validation data sets. **D.** Stage I patients in the validation data sets. **E.** Stage I patients in the combined discovery and validation data sets. **F.** Stage IV patients in the combined discovery and validation data sets. Each group was classified by CRC-113 gene signature into low and high risk, and evaluated by Kaplan-Meir analyses. *p* values were computed by log-rank test.

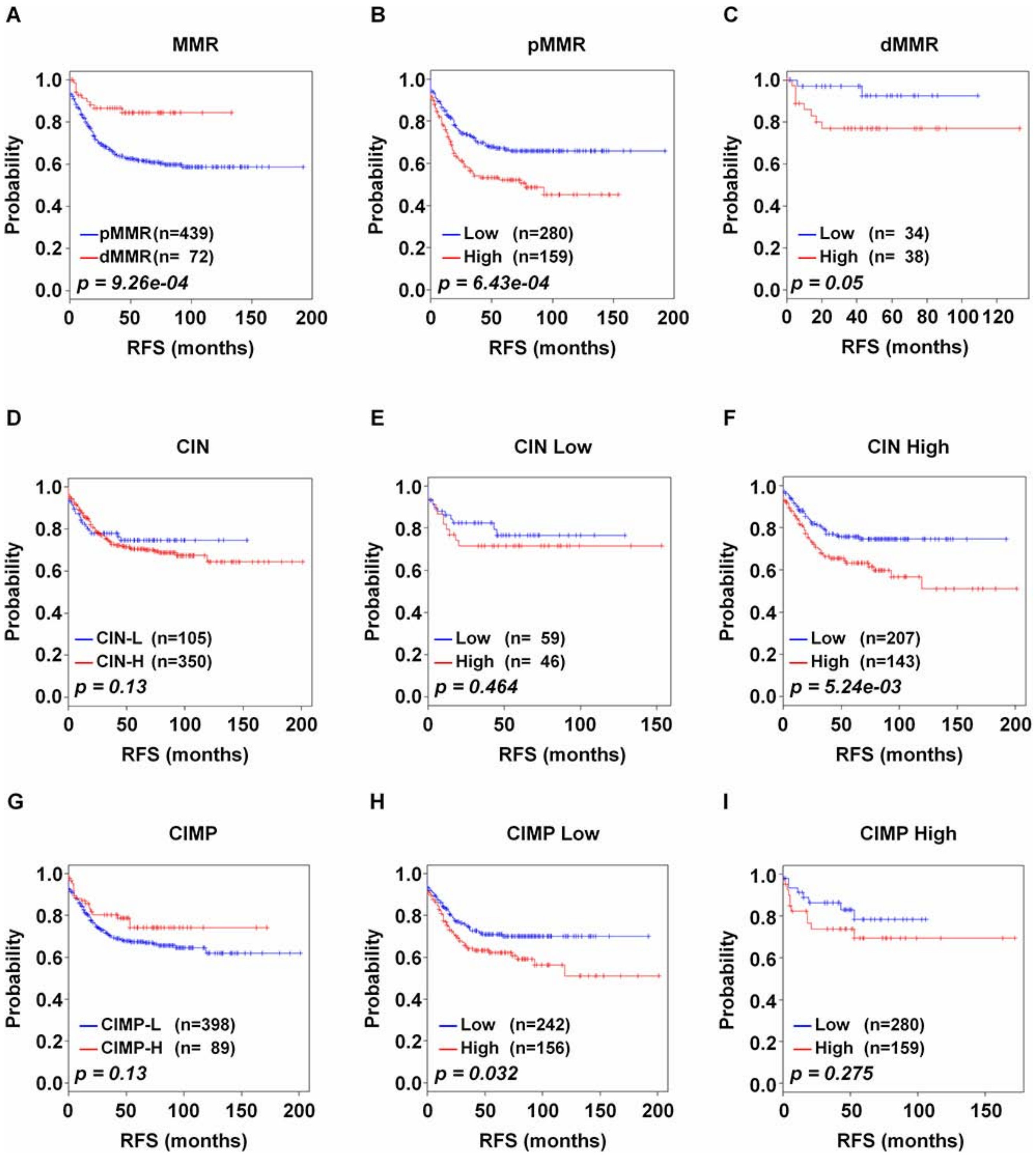

Supplementary Figure S5: Kaplan-Meier survival analysis of CRC-113 gene signature with CRC molecular pathways. A. MMR status was classified into pMMR and dMMR risk groups. **B** and **D.** pMMR and dMMR were classified by CRC-113 gene signature into low and high risk, respectively. **D.** CIN status was classified into CIN-L and CIN-H risk groups. **E** and **F.** CIN-L and CIN-H were classified by CRC-113 gene signature into low and high risk, respectively. **G.** CIMP status was classified into CIMP-L and CIMP-H risk groups. (H and I) CIMP-L and CIMP-H were classified by CRC-113 gene signature into low and high risk, respectively. Each classification was evaluated by Kaplan-Meir analyses. *p* values were computed by log-rank test.

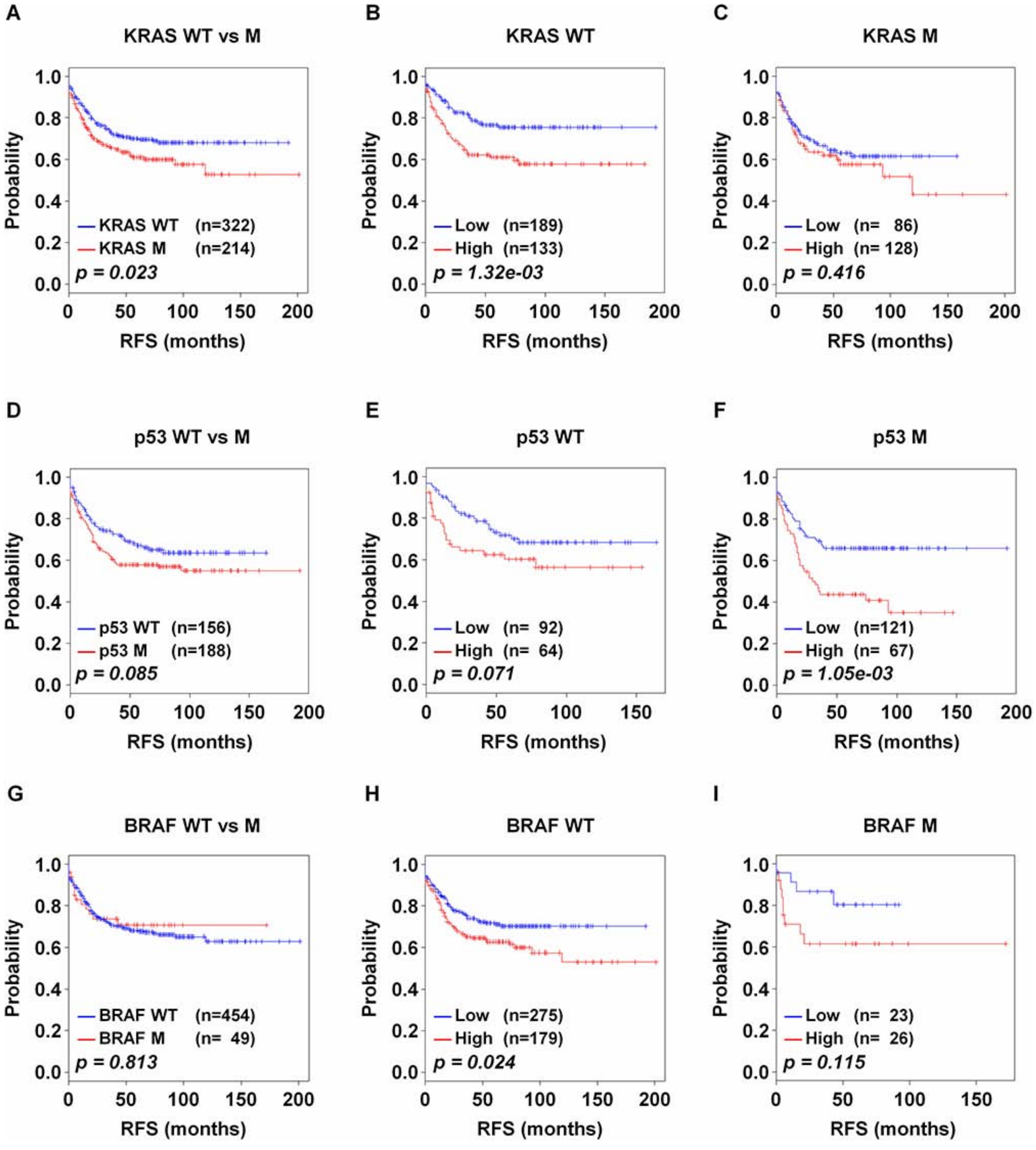

Supplementary Figure S6: Kaplan-Meier survival analysis of CRC-113 gene signature with gene mutations. A. KRAS status was classified into KRAS WT and M risk groups. **B** and **D.** KRAS WT and M were classified by CRC-113 gene signature into low and high risk, respectively. **D.** p53 status was classified into p53 WT and M risk groups. (E and F) p53 WT and M were classified by CRC-113 gene signature into low and high risk, respectively. **G.** BRAF status was classified into BRAF WT and M risk groups. **H** and **I.** BRAF WT and M were classified by CRC-113 gene signature into low and high risk, respectively. Each classification was evaluated by Kaplan-Meir analyses. *p* values were computed by log-rank test.

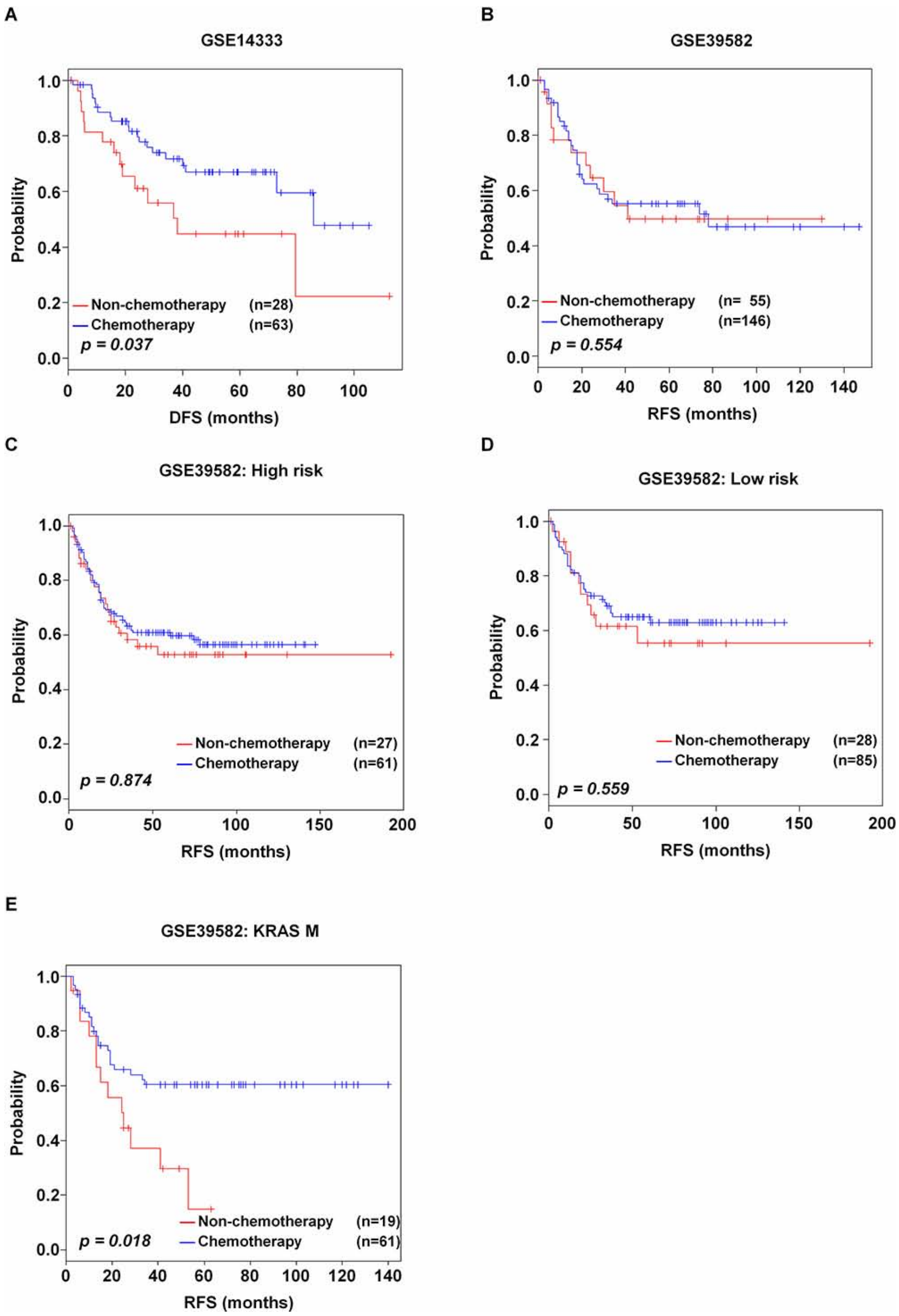

Supplementary Figure S7: Kaplan-Meier survival analysis of stage III CRC with adjuvant chemotherapy. **A** and **B.** GSE14333 and GSE39582 validation data sets, respectively. **C** and **D.** High risk and low risk patients in GSE39582 validation data set, respectively. **E.** Low risk patients with KRAS M in GSE39582 validation data set. Patients were separated according to chemotherapy treatment, and the chemotherapeutical advantage was evaluated by Kaplan-Meir analyses. *p* values were computed by log-rank test.

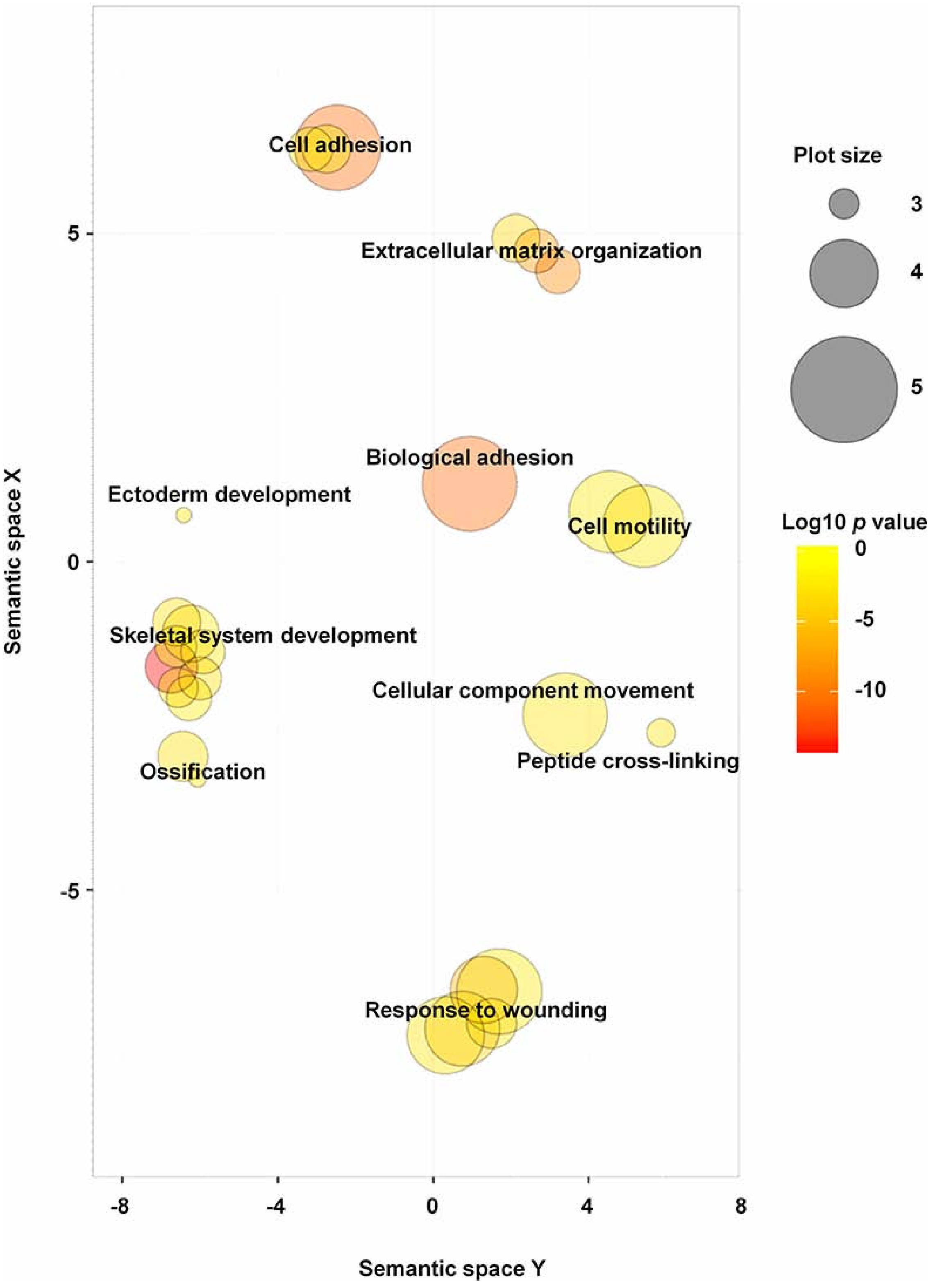

Supplementary Figure S8: GO term visualization (BP) for CRC-113 gene signature. The scatterplot shows the terms after redundancy reduction via REViGO, representing functional clusters. The bubble color indicates the *p*-values of the top-ranked GO enrichment analysis, and the bubble size indicates the frequency of the GO term in the underlying GO database. The GO terms cluster together in the semantic space according to functional similarity, without intrinsic meaning of semantic space units.

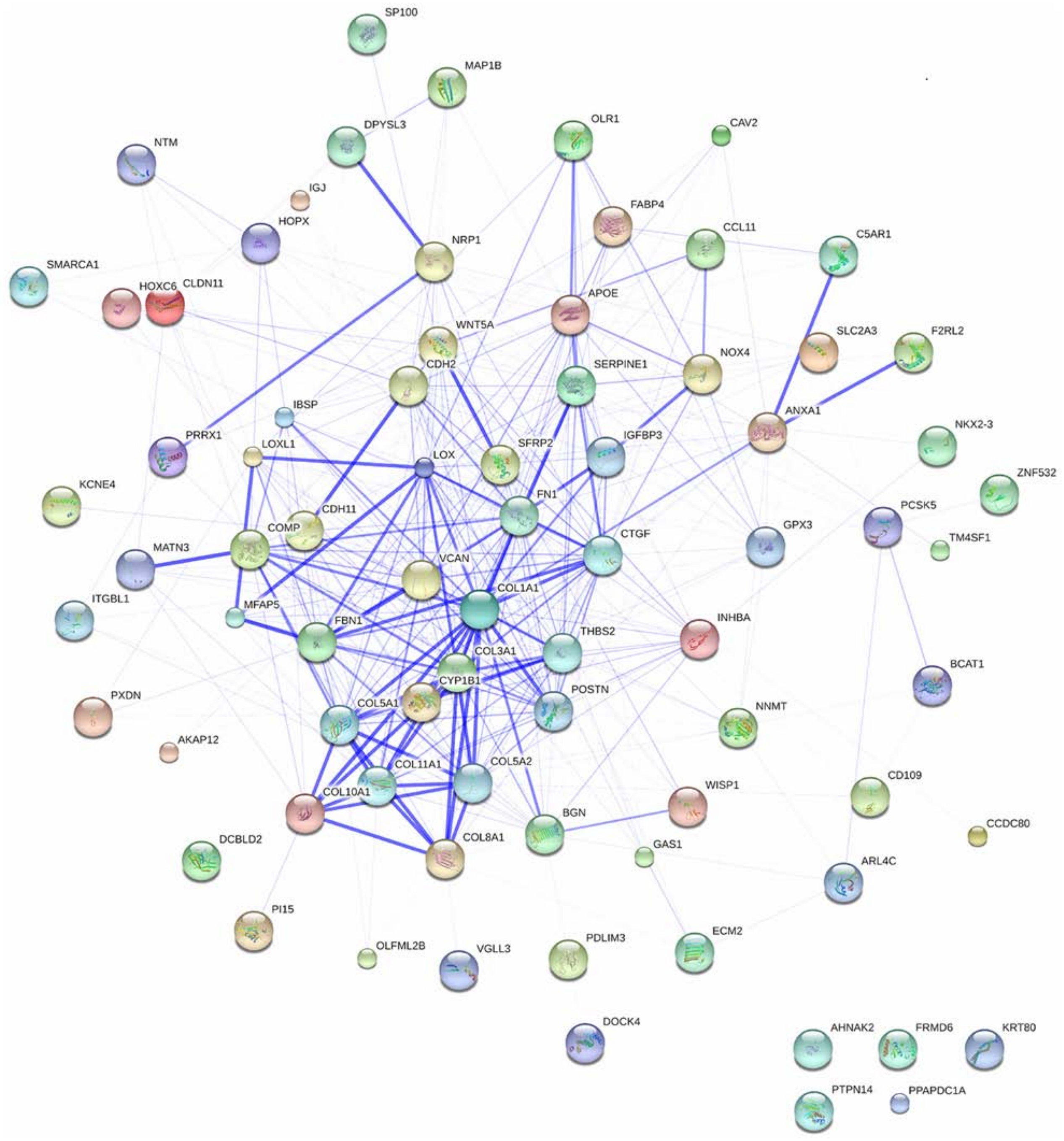

Supplementary Figure S9: Protein interaction network analysis in CRC-113 gene signature. Interaction map was generated using the STRING database.

Supplementary Table S1: Annotation of CRC-113 gene signature.

Symbol	Name	EntrezID	UniProtKB ID	Probe Set	Weighting Coefficient	HR	Jorrisen, et al (GSE14333)	De Sousa E Melo, et al (GSE33113)	Marisa, et al (GSE39582)	Oh, et al
AHNAK2	AHNAK nucleoprotein 2	113146	Q8IVF2	212992_at	0.005063	1.752				O
AKAP12	A kinase (PRKA) anchor protein 12	9590	Q02952	227529_s_at	0.005406	1.703	O		O	
				210517_s_at	0.005728	1.753	O		O	
ANXA1	annexin A1	301	P04083	201012_at	0.004248	1.901			O	O
APOE	apolipoprotein E	348	P02649	203382_s_at	0.006537	1.956			O	
				203381_s_at	0.006239	1.796			O	
ARL4C	ADP-ribosylation factor-like 4C	10123	P56559	202207_at	0.006604	1.871		O		
				202206_at	0.006354	1.865				
BCAT1	branched chain amino-acid transaminase 1, cytosolic	586	P54687	226517_at	0.007307	1.923			O	O
				225285_at	0.006161	2.037			O	
BGN	biglycan	633	P21810	213905_x_at	0.007932	2.021			O	
				201261_x_at	0.007022	2.196			O	
C5AR1	complement component 5a receptor 1	728	P21730	220088_at	0.006436	1.996			O	
CAV2	caveolin 2	858	P51636	203324_s_at	0.003326	2.036				
CCDC80	coiled-coil domain containing 80	151887	Q76M96	225242_s_at	0.00938	1.734		O	O	
CCL11	chemokine (C-C motif) ligand 11	6356	P51671	210133_at	-0.001598	0.351				
CD109	CD109 molecule	135228	Q6YHK3	226545_at	0.006661	1.527			O	O
CDH11	cadherin 11, type 2, OB-cadherin (osteoblast)	1009	P55287	236179_at	0.008771	1.648				

(*Continued*)

Symbol	Name	EntrezID	UniProtKB ID	Probe Set	Weighting Coefficient	HR	Jorrisen, et al (GSE14333)	De Sousa E Melo, et al (GSE33113)	Marisa, et al (GSE39582)	Oh, et al
CDH2	cadherin 2, type 1, N-cadherin (neuronal)	1000	P19022	203440_at	0.003323	2.577				
CLDN11	claudin 11	5010	O75508	228335_at	0.008699	1.444	O		O	
COL1A1	collagen, type I, alpha 1	1277	P02452	202310_s_at	0.005707	2.465			O	
COL3A1	collagen, type III, alpha 1	1281	P02461	232458_at	0.005892	1.774				
				215077_at	0.001218	5.103				
COL5A1	collagen, type V, alpha 1	1289	P20908	212488_at	0.006291	1.816	O		O	
COL5A2	collagen, type V, alpha 2	1290	P05997	221730_at	0.00636	1.881			O	
				221729_at	0.005891	2.068			O	
COL8A1	collagen, type VIII, alpha 1	1295	P27658	226237_at	0.010247	1.62	O		O	
COL10A1	collagen, type X, alpha 1	1300	Q03692	205941_s_at	0.010517	1.682	O			O
				217428_s_at	0.013833	1.539	O		O	O
COL11A1	collagen, type XI, alpha 1	1301	P12107	204320_at	0.009023	1.779				
				37892_at	0.011383	1.604				O
				229271_x_at	0.010746	1.509				O
COMP	cartilage oligomeric matrix protein	1311	P49747	205713_s_at	0.010628	1.437	O			
CTGF	connective tissue growth factor	1490	P29279	209101_at	0.00607	1.995				
CYP1B1	cytochrome P450, family 1, subfamily B, polypeptide 1	1545	Q16678	202437_s_at	0.016513	1.473			O	O
				202436_s_at	0.011542	1.612	O	O	O	O
				202435_s_at	0.0094	1.699	O		O	
DCBLD2	discoidin, CUB and LCCL domain containing 2	131566	Q96PD2	224911_s_at	0.004048	1.901	O			
				230175_s_at	0.002807	1.802	O			

(Continued)

Symbol	Name	EntrezID	UniProtKB ID	Probe Set	Weighting Coefficient	HR	Jorrisen, et al (GSE14333)	De Sousa E Melo, et al (GSE33113)	Marisa, et al (GSE39582)	Oh, et al
DOCK4	dedicator of cytokinesis 4	9732	Q8N1I0	205003_at	0.004025	2.465				
DPYSL3	dihydropyrimidinase-like 3	1809	Q14195	201430_s_at	0.005131	2.014			O	
ECM2	extracellular matrix protein 2, female organ and adipocyte specific	1842	O94769	206101_at	0.007733	1.725		O	O	
F2RL2	coagulation factor II (thrombin) receptor-like 2	2151	O00254	230147_at	-0.00203	0.386				
FABP4	fatty acid binding protein 4, adipocyte	2167	P15090	203980_at	0.007163	1.613			O	
FBN1	fibrillin 1	2200	P35555	235318_at	0.007537	1.751				
FN1	fibronectin 1	2335	P02751	211719_x_at	0.005375	3.45	O		O	
				210495_x_at	0.005736	3.005	O		O	
				216442_x_at	0.005853	2.928	O		O	
				212464_s_at	0.006046	2.79	O		O	
FRMD6	FERM domain containing 6	122786	Q96NE9	225481_at	0.007535	1.812			O	
				225464_at	0.006802	1.853		O	O	
GAS1	growth arrest-specific 1	2619	P54826	204457_s_at	0.009886	1.636		O	O	O
GPX3	glutathione peroxidase 3 (plasma)	2878	P22352	201348_at	0.005584	1.744				
HOPX	HOP homeobox	84525	Q9BPY8	211597_s_at	0.007347	1.764	O	O	O	
HOXC6	homeobox C6	3223	P09630	206858_s_at	0.00399	1.571		O	O	
IBSP	integrin-binding sialoprotein	3381	P21815	236028_at	0.007779	1.441				

(Continued)

Symbol	Name	EntrezID	UniProtKB ID	Probe Set	Weighting Coefficient	HR	Jorrisen, et al (GSE14333)	De Sousa E Melo, et al (GSE33113)	Marisa, et al (GSE39582)	Oh, et al
IGFBP3	insulin-like growth factor binding protein 3	3486	P17936	212143_s_at	0.004687	2.028				
IGJ	immunoglobulin J polypeptide, linker protein for immunoglobulin alpha and mu polypeptides	3512	P01591	212592_at	-0.0039	0.721	O			
INHBA	inhibin, beta A	3624	P08476	227140_at	0.007934	1.938		O		
				210511_s_at	0.007293	1.916	O			
ITGBL1	integrin, beta-like 1 (with EGF-like repeat domains)	9358	O95965	205422_s_at	0.012309	1.453	O			
KCNE4	potassium voltage-gated channel, Isk-related family, member 4	23704	Q8WWG9	222379_at	0.005136	2.034	O	O	O	
KRT80	keratin 80	144501	Q6KB66	231849_at	0.001886	2.256				
LOX	lysyl oxidase	4015	P28300	215446_s_at	0.008413	1.645				O
LOXL1	lysyl oxidase-like 1	4016	Q08397	203570_at	0.006334	2.042				
MAP1B	microtubule-associated protein 1B	4131	P46821	226084_at	0.006317	1.835		O		
MATN3	matrilin 3	4148	O15232	206091_at	0.011029	1.366				
MFAP5	microfibrillar associated protein 5	8076	Q13361	213765_at	0.010128	1.604				
				213764_s_at	0.013668	1.433				
				209758_s_at	0.011377	1.545				
NKX2–3	NK2 homeobox 3	159296	Q8TAU0	1553808_a_at	-0.001571	0.408			O	
NNMT	nicotinamide N-methyltransferase	4837	P40261	202237_at	0.006313	1.963		O		

(Continued)

Symbol	Name	EntrezID	UniProtKB ID	Probe Set	Weighting Coefficient	HR	Jorrisen, et al (GSE14333)	De Sousa E Melo, et al (GSE33113)	Marisa, et al (GSE39582)	Oh, et al
NOX4	NADPH oxidase 4	50507	Q9NPH5	219773_at	0.008308	1.806			O	
NRP1	neuropilin 1	8829	O14786	212298_at	0.006065	2.074			O	
OLFML2B	olfactomedin-like 2B	25903	Q68BL8	213125_at	0.006759	1.944	O		O	
OLR1	oxidized low density lipoprotein (lectin-like) receptor 1	4973	P78380	210004_at	0.009383	1.56	O			O
PCSK5	proprotein convertase subtilisin/kexin type 5	5125	Q92824	213652_at	0.004052	2.002				
PDLIM3	PDZ and LIM domain 3	27295	Q53GG5	209621_s_at	0.00656	1.857		O	O	
PI15	peptidase inhibitor 15	51050	O43692	229947_at	0.002931	2.19				
POSTN	periostin, osteoblast specific factor	10631	Q15063	210809_s_at	0.006128	2.344	O	O	O	
				1555778_a_at	0.008405	1.782	O			O
PPAPDC1A	phosphatidic acid phosphatase type 2 domain containing 1A	196051	Q5VZY2	236044_at	0.006179	1.807				
PRRX1	paired related homeobox 1	5396	P54821	226695_at	0.00788	1.743		O	O	
				238852_at	0.00823	1.561				
				205991_s_at	0.006395	1.815				
PTPN14	protein tyrosine phosphatase, non-receptor type 14	5784	Q15678	242321_at	0.002812	2.687				
PXDN	peroxidasin homolog (Drosophila)	7837	Q92626	212013_at	0.004444	2.046				
SERPINE1	serpin peptidase inhibitor, clade E (nexin, plasminogen activator inhibitor type 1), member 1	5054	P05121	202627_s_at	0.005189	2.018				

(Continued)

Symbol	Name	EntrezID	UniProtKB ID	Probe Set	Weighting Coefficient	HR	Jorrisen, et al (GSE14333)	De Sousa E Melo, et al (GSE33113)	Marisa, et al (GSE39582)	Oh, et al
SFRP2	secreted frizzled-related protein 2	6423	Q96HF1	223122_s_at	0.014999	1.641			O	O
				223121_s_at	0.013893	1.574			O	O
SLC2A3	solute carrier family 2 (facilitated glucose transporter), member 3	6515	P11169	202499_s_at	0.005542	1.72				
				222088_s_at	0.004853	1.783				
SMARCA1	SWI/SNF related, matrix associated, actin dependent regulator of chromatin, subfamily a, member 1	6594	P28370	203874_s_at	0.004765	2.318				
				203875_at	0.003645	2.375				
				215294_s_at	0.00438	1.912				
SP100	SP100 nuclear antigen	6672	P23497	202863_at	0.002155	2.901			O	
THBS2	thrombospondin 2	7058	P35442	203083_at	0.009165	2.072	O		O	
TM4SF1	transmembrane 4 L six family member 1	4071	P30408	215034_s_at	0.002248	2.604				
				209387_s_at	0.002221	2.456				
LOC100288985	Transcribed locus			230746_s_at	0.005128	1.963				
LOC646324	Transcribed locus			229479_at	0.007797	1.685	O		O	
FLJ38472	Homo sapiens cDNA FLJ38472 fis, clone FEBRA2022148			232113_at	0.00427	2.267			O	
NTM	neurotrimin	50863	Q9P121	227566_at	0.00755	1.719	O			
VCAN	versican	1462	P13611	204619_s_at	0.007458	2.116	O		O	
				204620_s_at	0.006814	2.212		O	O	
				221731_x_at	0.006671	2.197			O	
				215646_s_at	0.008676	1.761			O	
				211571_s_at	0.00805	1.755			O	
VGLL3	vestigial like 3 (Drosophila)	389136	A8MV65	227399_at	0.009256	1.748		O		O

(Continued)

Symbol	Name	EntrezID	UniProtKB ID	Probe Set	Weighting Coefficient	HR	Jorrisen, et al (GSE14333)	De Sousa E Melo, et al (GSE33113)	Marisa, et al (GSE39582)	Oh, et al
WISP1	WNT1 inducible signaling pathway protein 1	8840	O95388	229802_at	0.00748	1.785				
				235821_at	0.004712	2.141				
WNT5A	wingless-type MMTV integration site family, member 5A	7474	P41221	205990_s_at	-0.002052	0.535				
ZNF532	zinc finger protein 532	55205	Q9HCE3	225021_at	0.004879	2.315	O			

HR, hazard ratio

Supplementary Table S2: Clinicopathological characteristics of CRC patients in two risk groups of the GSE14333 data set.

Variable		Total	Low-risk	High-risk	p (χ^2- test)
**Number of patients (%)**		**226**	**139**	**87**	
**Gender**	**Male**	120 (53.1)	77 (55.4)	43 (49.4)	0.056
	**Female**	106 (46.9)	62 (44.6)	44 (50.6)	
**Age**	**<70**	125 (55.3)	72 (51.8)	53 (60.9)	0.136
	**≥70**	101 (44.7)	67 (48.2)	34 (39.1)	
**Duke's Stage**	**A**	41 (18.1)	34 (24.5)	7 (8.0)	2.98e-59
	**B**	94 (41.6)	58 (41.7)	36 (41.4)	
	**C**	91 (40.6)	91 (40.6)	44 (50.6)	
	**D**	0	0	0	
**location**	**Colon**	2 (0.9)	2 (1.4)	0	0.442
	**Rectum**	30 (13.3)	18 (12.9)	12 (13.8)	
	**Left**	93 (41.2)	52 (37.4)	41 (47.1)	
	**Right**	101 (44.7)	67 (48.2)	34 (39.1)	
**DFS**	**0**	176 (77.9)	121 (87.1)	55 (63.2)	4.57e-09
	**1**	50 (22.1)	18 (12.9)	32 (36.8)	

DFS, disease free survival; p values were obtained from the χ^2 -test.

Supplementary Table S3: Clinicopathological characteristics of CRC patients in two risk groups of the GSE33113 data set.

Variable		Total	Low-risk	High-risk	p (χ^2- test)
**Number of patients (%)**		**90**	**60**	**30**	
**Gender**	**Male**	42 (46.7)	31 (51.7)	11 (36.7)	0.652
	**Female**	48 (53.3)	29 (48.3)	19 (63.3)	
**Age**	**<70**	39 (43.3)	25 (41.7)	14 (46.7)	0.179
	**≥70**	51 (56.7)	35 (58.3)	16 (53.3)	
**RFS**	**0**	71 (78.9)	52 (86.7)	19 (63.3)	0.011
	**1**	19 (21.1)	8 (13.3)	11 (36.7)	

RFS, recurrence free survival; p values were obtained from the χ^2 -test.

Supplementary Table S4: Clinicopathological characteristics of CRC patients in two risk groups of the GSE39582 data set.

Variable		Total	Low-risk	High-risk	*p* (χ^2- test)
**Number of patients (%)**		**557**	**331**	**226**	
**Gender**	**Male**	250 (44.9)	147 (44.4)	103 (45.6)	0.427
	**Female**	307 (55.1)	184 (55.6)	123 (54.4)	
**Age**	**<70**	299 (53.7)	177 (53.5)	122 (54.0)	0.488
	**≥70**	258 (46.3)	154 (46.5)	104 (46.0)	
**AJCC stage**	**0**	4 (0.7)	4 (1.2)	0 (0.0)	0.034
	**I**	32 (5.7)	26 (7.9)	6 (2.7)	
	**II**	260 (46.7)	155 (46.8)	105 (46.5)	
	**III**	201 (36.1)	113 (34.1)	88 (38.9)	
	**IV**	60 (10.8)	33 (10.0)	27 (11.9)	
**Location**	**Distal**	339 (60.9)	212 (64.0)	127 (56.2)	0.062
	**Proximal**	218 (39.1)	119 (36.0)	99 (43.8)	
**RFS**	**0**	380 (68.2)	241 (72.8)	139 (61.5)	3.00e-03
	**1**	177 (31.8)	90 (27.2)	87 (38.5)	

AJCC, American Joint Committee on Cancer; RFS, relapse free survival; *p* values were obtained from the χ^2 -test.

Supplementary Table S5: Univariate and multivariate Cox proportional hazard regression analyses of clinical variables in the GSE14333 data set.

Variable	Univariate analysis			Multivariate analysis		
	HR	95% CI	*p* value	HR	95% CI	*p* value
**Gender**	0.908	0.520–1.588	0.736	1.019	0.5667–1.831	0.950
**Age**	0.981	0.961–1.001	0.069	0.989	0.968–1.011	0.317
**Dukes' Stage**	2.949	1.808–4.812	1.48e-05	2.548	1.537–4.226	2.89e-04
**Location**	0.829	0.558–1.232	0.354	0.967	0.636–1.469	0.874
**Chemotherapy**	0.529	0.303–0.922	0.025	1.428	0.726–2.809	0.301
**Risk**	2.898	1.625–5.167	3.12e-04	2.180	1.212–3.920	9.27e-03

HR, hazard ratio; CI, Confidence Interval; *p* values were obtained from the χ^2 -test.

Supplementary Table S6: Univariate and multivariate Cox proportional hazard regression analyses of clinical variables in the GSE33113 data set.

Variable	Univariate analysis			Multivariate analysis		
	HR	95% CI	*p* value	HR	95% CI	*p* value
**Gender**	1.128	0.454–2.807	0.795	0.983	0.944–1.023	0.39
**Age**	0.984	0.951–1.019	0.366	1.069	0.410–2.790	0.891
**Risk**	3.289	1.320–8.190	0.011	3.177	1.269–7.953	0.014

HR, hazard ratio; CI, Confidence Interval; *p* values were obtained from the χ^2 -test.

Supplementary Table S7: Univariate and multivariate Cox proportional hazard regression analyses of clinical variables in the GSE39582 data set.

Variable	Univariate analysis			Multivariate analysis		
	HR	95% CI	*P* value	HR	95% CI	*P* value
**Gender**	0.889	0.765–1.033	0.124	0.681	0.498–0.931	0.016
**Age**	0.999	0.988–1.011	0.910	1.007	0.995–1.020	0.253
**AJCC Stage**	2.605	2.109–3.218	6.79e-19	2.243	1.716–2.931	3.36e-09
**Chemotherapy**	0.553	0.405–0.745	1.84e-04	0.977	0.675–1.414	0.902
**Location**	1.288	0.943–1.758	0.111	1.248	0.875–1.779	0.222
**MMR**	0.498	0.263–0.946	0.033	0.491	0.234–1.031	0.060
**CIMP**	1.447	0.893–2.346	0.134	1.422	0.697–2.900	0.333
**CIN**	0.883	0.565–1.381	0.585	1.402	0.844–2.330	0.192
**KRAS**	1.416	1.046–1.916	0.024	1.517	1.084–2.122	0.015
**BRAF**	0.932	0.528–1.647	0.809	1.667	0.692–4.013	0.255
**p53**	1.358	0.955–1.931	0.088	1.124	0.773–1.634	0.542
**Risk**	1.535	1.143–2.061	4.40e-03	1.674	1.237–2.264	8.37e-04

AJCC, American Joint Committee on Cancer; DFS, disease free survival; DSS, disease specific survival; OS, overall survival; HR, hazard ratio; CI, Confidence Interval; *p* values were obtained from the χ^2 -test.

Supplementary Table S8: Molecular characteristics of CRC patients in two risk groups of the GSE39582 data set.

Variable	Status	Risk	RFS total		RFS 0		RFS 1		p (χ^2-test)
MMR	Deficient	Low	34	6.65%	32	6.26%	2	0.39%	1.33e-05
		High	38	7.44%	30	5.87%	8	1.57%	
	Proficient	Low	280	54.79%	195	38.16%	85	16.63%	
		High	159	31.12%	87	17.03%	72	14.09%	
	Total		511	100.00%	344	67.32%	167	32.68%	
CIN	Low	Low	59	12.97%	47	10.33%	12	2.64%	0.035
		High	46	10.11%	34	7.47%	12	2.64%	
	High	Low	207	45.49%	161	35.38%	46	10.11%	
		High	143	31.43%	92	20.22%	51	11.21%	
	Total		455	100.00%	334	73.41%	121	26.59%	
CIMP	Low	Low	242	49.69%	177	36.34%	65	13.35%	0.045
		High	156	32.03%	98	20.12%	58	11.91%	
	High	Low	47	9.65%	39	8.01%	8	1.64%	
		High	42	8.62%	31	6.37%	11	2.26%	
	Total		487	100.00%	345	70.84%	142	29.16%	
KRAS	WT	Low	189	35.26%	148	27.61%	41	7.65%	1.91e-03
		High	133	24.81%	83	15.49%	50	9.33%	
	M	Low	128	23.88%	84	15.67%	44	8.21%	
		High	86	16.04%	52	9.70%	34	6.34%	
	Total		536	100.00%	367	68.47%	169	31.53%	
p53	WT	Low	92	26.74%	66	19.19%	26	7.56%	3.88e-04
		High	64	18.60%	39	11.34%	25	7.27%	
	M	Low	121	35.17%	81	23.55%	40	11.63%	
		High	67	19.48%	28	8.14%	39	11.34%	
	Total		344	100.00%	214	62.21%	130	37.79%	
BRAF	WT	Low	275	54.67%	203	40.36%	72	14.31%	0.053
		High	179	35.59%	114	22.66%	65	12.92%	
	M	Low	23	4.57%	19	3.78%	4	0.80%	
		High	26	5.17%	17	3.38%	9	1.79%	
	Total		503	100.00%	353	70.18%	150	29.82%	

RFS, relapse free survival; MMR, DNA mismatch repair; CIN, chromosomal instability;l CIMP, CpG-island methylator phenotype (CIMP); WT, wild type; M, mutant; *p* values were obtained from the χ^2 -test.

Supplementary Table S9: List of significant GO terms (biological process).

GO Term	Description	*p* value	Benjamini	Count	Percent (%)	Genes
**GO:0001501**	skeletal system development	5.77E-15	5.57E-12	19	24.68	IBSP, WNT5A, MATN3, FBN1, COL3A1, PRRX1, POSTN, GAS1, COL5A2, HOXC6, INHBA, CTGF, COMP, COL1A1, IGFBP3, COL11A1, PCSK5, COL10A1, CDH11
**GO:0007507**	heart development	7.40E-05	5.47E-03	8	10.39	NRP1, FBN1, COL3A1, HOPX, PDLIM3, COL11A1, PCSK5, COL5A1
**GO:0001568**	blood vessel development	2.39E-06	2.88E-04	10	12.99	BGN, NRP1, CTGF, APOE, COL3A1, PRRX1, COL1A1, LOX, CDH2, COL5A1
**GO:0001944**	vasculature development	2.92E-06	3.12E-04	10	12.99	BGN, NRP1, CTGF, APOE, COL3A1, PRRX1, COL1A1, LOX, CDH2, COL5A1
**GO:0007155**	cell adhesion	5.14E-12	2.48E-09	22	28.57	DCBLD2, IBSP, NRP1, OLR1, COL3A1, POSTN, CDH2, CLDN11, ECM2, COL5A1, ITGBL1, CCL11, WISP1, CTGF, COMP, VCAN, COL8A1, THBS2, COL11A1, NTM, FN1, CDH11
**GO:0009611**	response to wounding	1.64E-07	2.63E-05	15	19.48	DCBLD2, NOX4, F2RL2, NRP1, OLR1, COL3A1, MAP1B, ANXA1, COL5A1, CCL11, CTGF, SERPINE1, VCAN, LOX, FN1
**GO:0018149**	peptide cross-linking	2.54E-04	1.28E-02	4	5.19	BGN, COL3A1, ANXA1, FN1
**GO:0022610**	biological adhesion	5.28E-12	1.70E-09	22	28.57	DCBLD2, IBSP, NRP1, OLR1, COL3A1, POSTN, CDH2, CLDN11, ECM2, COL5A1, ITGBL1, CCL11, WISP1, CTGF, COMP, VCAN, COL8A1, THBS2, COL11A1, NTM, FN1, CDH11
**GO:0030198**	extracellular matrix organization	1.48E-09	2.85E-07	10	12.99	IBSP, COL3A1, CCDC80, POSTN, COL1A1, LOX, ECM2, COL5A2, COL11A1, COL5A1
**GO:0030199**	collagen fibril organization	2.38E-07	3.28E-05	6	7.79	COL3A1, COL1A1, LOX, COL5A2, COL11A1, COL5A1
**GO:0048870**	cell motility	6.53E-04	2.15E-02	8	10.39	CAV2, NRP1, CTGF, VCAN, CDH2, COL5A1, FN1, NKX2–3
**GO:0016477**	cell migration	3.45E-04	1.50E-02	8	10.39	CAV2, NRP1, CTGF, VCAN, CDH2, COL5A1, FN1, NKX2–3
**GO:0006928**	cell motion	4.18E-04	1.60E-02	10	12.99	CAV2, NRP1, CTGF, ANXA1, VCAN, GAS1, CDH2, COL5A1, FN1, NKX2–3
**GO:0051674**	localization of cell	6.53E-04	2.15E-02	8	10.39	CAV2, NRP1, CTGF, VCAN, CDH2, COL5A1, FN1, NKX2–3

(*Continued*)

GO Term	Description	*p* value	Benjamini	Count	Percent (%)	Genes
**GO:0043062**	extracellular structure organization	2.83E-10	6.81E-08	12	15.58	IBSP, COL3A1, MAP1B, CCDC80, POSTN, COL1A1, LOX, CDH2, ECM2, COL5A2, COL11A1, COL5A1
**GO:0001558**	regulation of cell growth	3.86E-05	3.09E-03	8	10.39	DCBLD2, INHBA, WISP1, NRP1, CTGF, APOE, MAP1B, IGFBP3
**GO:0048638**	regulation of developmental growth	1.48E-03	3.78E-02	4	5.19	NRP1, APOE, MAP1B, HOPX
**GO:0040008**	regulation of growth	2.17E-04	1.38E-02	9	11.69	DCBLD2, INHBA, WISP1, NRP1, CTGF, APOE, MAP1B, HOPX, IGFBP3
**GO:0042060**	wound healing	3.03E-04	1.45E-02	7	9.09	DCBLD2, F2RL2, SERPINE1, COL3A1, LOX, COL5A1, FN1
**GO:0007398**	ectoderm development	3.77E-04	1.50E-02	7	9.09	CTGF, COL3A1, ANXA1, AHNAK2, COL1A1, COL5A2, COL5A1
**GO:0000302**	response to reactive oxygen species	2.92E-05	2.81E-03	6	7.79	PXDN, OLR1, APOE, GPX3, SERPINE1, COL1A1
**GO:0032964**	collagen biosynthetic process	2.25E-04	1.27E-02	3	3.90	COL3A1, COL1A1, COL5A1
**GO:0006979**	response to oxidative stress	1.13E-03	3.25E-02	6	7.79	PXDN, OLR1, APOE, GPX3, SERPINE1, COL1A1
**GO:0001503**	ossification	2.24E-04	1.34E-02	6	7.79	IBSP, CTGF, COL1A1, COL5A2, IGFBP3, CDH11
**GO:0007160**	cell-matrix adhesion	8.73E-04	2.77E-02	5	6.49	CTGF, COL3A1, ECM2, FN1, ITGBL1
**GO:0010810**	regulation of cell-substrate adhesion	1.39E-03	3.65E-02	4	5.19	CCDC80, COL1A1, ECM2, COL8A1
**GO:0060173**	limb development	1.50E-03	3.74E-02	5	6.49	WNT5A, COMP, PRRX1, GAS1, PCSK5
**GO:0048736**	appendage development	1.50E-03	3.74E-02	5	6.49	WNT5A, COMP, PRRX1, GAS1, PCSK5
**GO:0031589**	cell-substrate adhesion	1.25E-03	3.48E-02	5	6.49	CTGF, COL3A1, ECM2, FN1, ITGBL1

(*Continued*)

GO Term	Description	*p* value	Benjamini	Count	Percent (%)	Genes
**GO:0048706**	embryonic skeletal system development	3.31E-05	2.90E-03	6	7.79	HOXC6, PRRX1, COL1A1, GAS1, COL11A1, PCSK5
**GO:0043009**	chordate embryonic development	1.02E-03	3.11E-02	8	10.39	HOXC6, SFRP2, HOPX, PRRX1, COL1A1, GAS1, COL11A1, PCSK5
**GO:0048705**	skeletal system morphogenesis	2.04E-03	4.81E-02	5	6.49	CTGF, PRRX1, COL1A1, GAS1, COL11A1
**GO:0051216**	cartilage development	4.34E-04	1.60E-02	5	6.49	WNT5A, CTGF, PRRX1, COL1A1, COL11A1
**GO:0060348**	bone development	3.06E-04	1.39E-02	6	7.79	IBSP, CTGF, COL1A1, COL5A2, IGFBP3, CDH11
**GO:0009792**	embryonic development ending in birth or egg hatching	1.07E-03	3.17E-02	8	10.39	HOXC6, SFRP2, HOPX, PRRX1, COL1A1, GAS1, COL11A1, PCSK5
**GO:0030323**	respiratory tube development	1.27E-04	8.74E-03	6	7.79	WNT5A, CAV2, CTGF, HOPX, LOX, PCSK5
**GO:0030324**	lung development	1.30E-03	3.51E-02	5	6.49	WNT5A, CAV2, CTGF, HOPX, LOX
**GO:0060541**	respiratory system development	1.79E-03	4.33E-02	5	6.49	WNT5A, CAV2, CTGF, HOPX, LOX
**GO:0010035**	response to inorganic substance	4.42E-04	1.57E-02	7	9.09	PXDN, OLR1, APOE, GPX3, SERPINE1, MAP1B, COL1A1
**GO:0035295**	tube development	6.42E-04	2.19E-02	7	9.09	WNT5A, CAV2, NRP1, CTGF, HOPX, LOX, PCSK5
**GO:0008544**	epidermis development	2.48E-04	1.32E-02	7	9.09	CTGF, COL3A1, ANXA1, AHNAK2, COL1A1, COL5A2, COL5A1
**GO:0043588**	skin development	3.54E-04	1.47E-02	4	5.19	COL3A1, COL1A1, COL5A2, COL5A1

p values were obtained from the χ^2 -test.
